# The effects of cytosine methylation on general transcription factors

**DOI:** 10.1038/srep29119

**Published:** 2016-07-07

**Authors:** Jianshi Jin, Tengfei Lian, Chan Gu, Kai Yu, Yi Qin Gao, Xiao-Dong Su

**Affiliations:** 1 Biodynamic Optical Imaging Center (BIOPIC), School of Life Sciences, Peking University, Beijing, China; 2 State Key Laboratory of Protein and Plant Gene Research, Peking University, Beijing, China; 3 Institute of Theoretical and Computational Chemistry, College of Chemistry and Molecular Engineering, Peking University, Beijing, China

## Abstract

DNA methylation on CpG sites is the most common epigenetic modification. Recently, methylation in a non-CpG context was found to occur widely on genomic DNA. Moreover, methylation of non-CpG sites is a highly controlled process, and its level may vary during cellular development. To study non-CpG methylation effects on DNA/protein interactions, we have chosen three human transcription factors (TFs): glucocorticoid receptor (GR), brain and muscle ARNT-like 1 (BMAL1) - circadian locomotor output cycles kaput (CLOCK) and estrogen receptor (ER) with methylated or unmethylated DNA binding sequences, using single-molecule and isothermal titration calorimetry assays. The results demonstrated that these TFs interact with methylated DNA with different effects compared with their cognate DNA sequences. The effects of non-CpG methylation on transcriptional regulation were validated by cell-based luciferase assay at protein level. The mechanisms of non-CpG methylation influencing DNA-protein interactions were investigated by crystallographic analyses and molecular dynamics simulation. With BisChIP-seq assays in HEK-293T cells, we found that GR can recognize highly methylated sites within chromatin in cells. Therefore, we conclude that non-CpG methylation of DNA can provide a mechanism for regulating gene expression through directly affecting the binding of TFs.

DNA methylation at the C_5_ position of cytosine (mC) plays important roles in many epigenetic processes, such as mammalian development, retrotransposon silencing and cellular reprogramming^1^,^2^. Previous detection methods have shown that high methylation levels are found mostly in the context of CpG (mCG). However, with the application of next-generation sequencing (NGS) single-base resolution technology to the whole genomic methylome^3^,^4^, the methylation of non-CpG moieties (mCH, where H = A, T or C) was recently found to be abundant and functionally significant, especially in embryonic stem cells^5^,^6^,^7^, induced pluripotent stem cells^8^, oocytes^9^ and neurons in the adult mammalian brain^10^,^11^,^12^,^13^. Furthermore, the level of non-CpG methylation varies during cellular development, e.g., methylation level decreases after the resumption of mitosis in the neonatal period and increases during brain development^10^,^14^. Methylation of non-CpG sites is a highly controlled process, but how non-CpG methylation controls DNA/protein interactions and regulates transcription is largely unknown^15^,^16^.

Although the methylation level of DNA is lower in the transcription promoter region than that of the gene body region^5^, the methylation in the promoter region could well participate in transcriptional regulation. The current models concerning gene regulation by DNA methylation are mostly repressive^17^. A typical model is when methylated DNA (mCG) binds to methylation binding proteins (MBPs), the binding of transcription factors (TFs) would be blocked and gene transcription would remain in the silent state^18^. Very recently, Gabel *et al*. and Chen *et al*. identified that methyl-CpG binding protein 2 (MeCP2) also has high binding affinity to mCH (especially to mCA) sites^19^,^20^,^21^, suggesting that non-CpG methylated sites can be recognized and bound by a specific protein as well. Since there is no special binding mode revealed by structural studies between MBPs and methylated DNA^22^,^23^,^24^,^25^,^26^, it is obvious that other DNA binding proteins, including many TFs may also have a chance to gain altered binding capacity (enhanced or weakened) for methylated DNA sequences, using the mC as a fifth base. However, recent structural and biochemical studies have mostly paid attention to the binding of MBPs^19^,^20^,^22^,^23^,^24^,^25^,^26^ or the nucleosome positioning on methylated DNA^27^,^28^,^29^. The few cases concerning the TFs binding to methylated DNA sequences directly only focused on CpG sites^30^.

In this study, we focus on the detailed interactions of three important human TFs: glucocorticoid receptor (GR), brain and muscle ARNT-like 1 (BMAL1) - circadian locomotor output cycles kaput (CLOCK), and estrogen receptor (ER) with their methylated or unmethylated DNA binding sequences, to elucidate general principles of structure-function relationship of DNA/protein interactions with methylated DNA. The results demonstrated that these TFs represent three possible mechanisms for regulating gene expression by DNA methylation respectively: GR represents the TF with binding affinity enhanced by the methylation; whereas BMAL1-CLOCK represents the TF with binding affinity weakened by the methylation; and there is yet another group of TFs, such as ER without significant affinity change towards the methylated DNA.

## Results

### Methylated DNA interacts with DNA binding proteins

**Figure 1:**
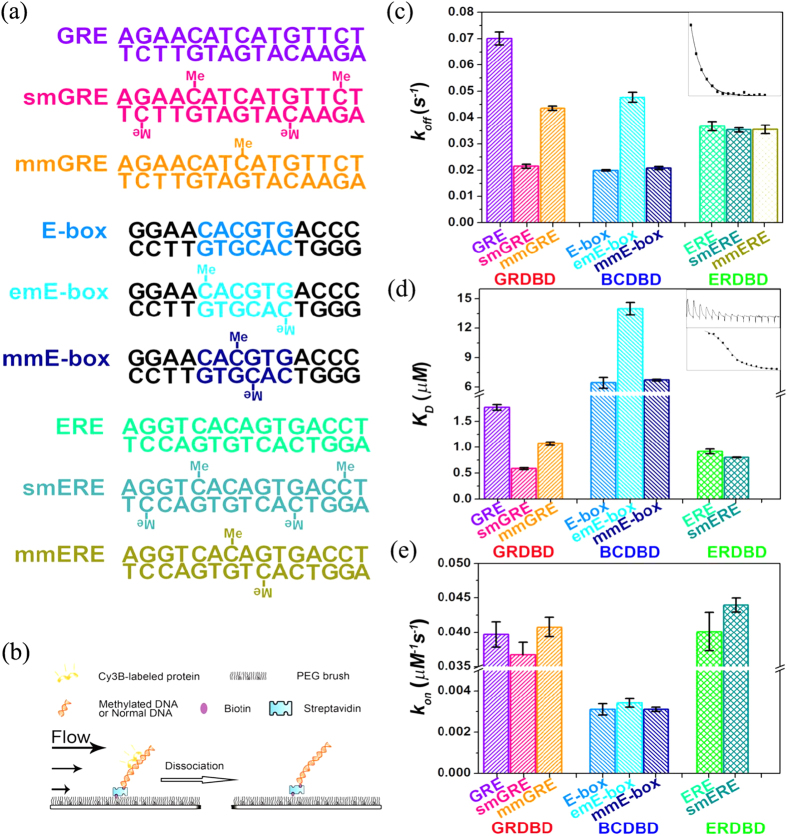
Affinity and binding stability of DNA/protein interactions are changed by the methylation of non-CpG context. **(a)** Constructs of the unmethylated GRE, E-box and ERE, methylated smGRE, mmGRE, emE-box, mmE-box, smERE and mmERE. The only methylated cytosine in the possible base steps that we have not studied is the latter cytosine in the CC context of ERE, which is very rare in the human genome (Supplementary Fig. S1). **(b)** Schematic of the single-molecule assay in a flow cell^31^. We measured the stochastic time of Cy3b-labeled protein dissociating from DNA. The DNA is methylated or unmethylated. **(c)** Dissociation rates, *k*_*off*_, of the GRDBD, BCDBD and ERDBD interacting with their methylated or unmethylated DNA binding targets. The rates were obtained by fitting the stochastic residence time to a single-exponential decay, and extracting the decay constant (insert and Supplementary Fig. S2), error bars denote the standard deviation of the fitting. **(d)** Dissociation constants *K*_D_ of the GRDBD, BCDBD and ERDBD interacting with their methylated or unmethylated DNA binding targets measured by ITC (insert and Supplementary Fig. S4). The final *K*_D_ is the average of three independent titrations and error bars denote the standard deviation. **(e)** Association constant *k*_*on*_ was calculated by *k*_*off*_/*K*_D_ and error bars denote the standard deviation from three individual experiments.

The dissociation rates (*k*_off_) of GR, BMAL1-CLOCK and ER towards their methylated or unmethylated DNA binding targets (Fig. 1a) were measured using their respective DNA binding domains (GRDBD, BCDBD and ERDBD) by a high-throughput single molecule assay (Fig. 1b)^31^. Both GRDBD and ERDBD belong to the zinc-finger family of DNA binding proteins and bind to GR regulation elements (GRE)^32^ and ER regulation elements (ERE)^33^, respectively. According to the crystal structures^32^,^33^, every monomer of GRDBD or ERDBD interacts with only hexametric half sites (6 bp) on both binding sites separated by 3 to 4 bp spacer. The conserved hexametric half site of GRE and ERE are “AGAACA” and “AGGTCA” respectively, and we used previously relevant sequences AGAACATCATGTTCT (GRE) and AGGTCACAGTGACCT (ERE)^32^,^33^ in this study.

We classified cytosines in GRE or ERE into two categories, one is in the hexametric half site (smGRE and smERE, sm for side-methylated); the other category is the spacer sequences (mmGRE and mmERE, mm for middle-methylated. The only cytosine we did not investigate here is the latter cytosine in the TGACCT context of ERE sequences, this site is rare in human genome. Supplementary Fig. S1). The BCDBD is a member of the basic helix-loop-helix family of DNA binding proteins and binds to the E-box with the palindromic “CACGTG” sequence^34^. We also classified E-box’s cytosines into two categories, because it has both non-CpG site (in the ends of both strands) and CpG site (in the middle of the CACGTG sequence), emE-box (em for end-methylated) and mmE-box (mm for middle-methylated).

The results of measured dissociation rates (*k*_*off*_) are presented in Fig. 1c and Supplementary Fig. S2. Interestingly, the changes to *k*_*off*_ as a result of DNA methylation for the three DNA binding proteins are distinctly different. The *k*_*off*_ of GRDBD binding to smGRE (side-methylated GRE) with four mCHs in the direct interacting region^32^,^35^, decreased by ~3 fold (Fig. 1c, magenta), whereas binding to mmGRE (middle-methylated GRE) with only one mCH in the middle of the GRE, decreased by ~1.5 fold (Fig. 1c, orange) compared with the binding to unmethylated GRE (Fig. 1c, violet). In both cases, the methylation of GRE increases the stability of the GRE-GRDBD complex, this is very different from the previous observation that GR has decreased binding affinity to its methylated binding site^30^. In their case, only the methylated CpG sites were considered, however, typical GR binding sites do not have CpG site^35^. In contrast, the *k*_*off*_ of BCDBD binding to the emE-box with mCHs at the end of the E-box, increased by ~2 fold (Fig. 1c, cyan), whereas binding to the mmE-box with mCGs in the middle of E-box, kept unchanged (Fig. 1c, navy blue) compared with its binding to the unmethylated E-box (Fig. 1c, azure). Therefore, methylation of the cytosine in CH context of the E-box decreases the stability of the E-box-BCDBD complex. Finally, the mCH does not affect the *k*_*off*_ of ERDBD dissociating from ERE (Fig. 1c, spring green, teal and olive).

The dissociation constants, *K*_D_, of the above complexes were measured using conventional ITC (isothermal titration calorimetry) and the results are presented in Fig. 1d and Supplementary Fig. S4. The differences in the *K*_D_ values are in good agreement with the *k*_*off*_ values, i.e., the methylation of GRE decreases the binding affinity of GRDBD, and the methylation of the CH context in the E-box increases the binding affinity of BCDBD. The methylations of E-box at the CpG contexts and ERE at the non-CpG contexts have negligible effects on the binding affinity of BCDBD and ERDBD.

The *k*_*on*_ was calculated from the *K*_D_ and *k*_*off*_, and is presented in Fig. 1e. The results demonstrated that the methylation of DNA does not change the *k*_*on*_ of the proteins in general.

### Effects of methylation on gene regulation

**Figure 2:**
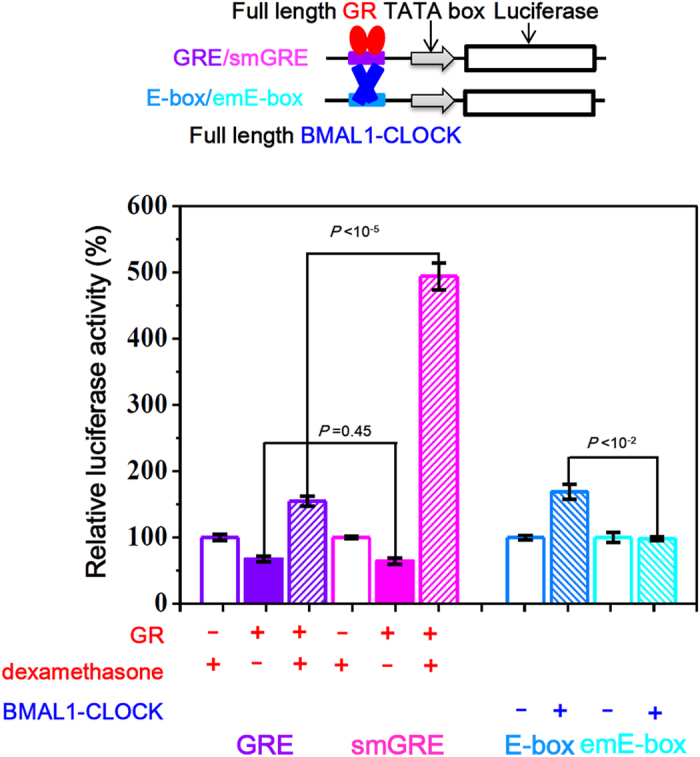
Cell-based luciferase assay revealed that enhanced or weakened binding of TFs to methylated DNA could affect gene expression. GRE/smGRE/E-box/emE-box was cloned upstream of TATA box in a SV40 promoter which drives a luciferase reporter gene (firefly luciferase). The activity of each non-activated system without protein expressed was set as 100%, and the relative luciferase activity of each system were presented by percentage of their non-activated control. Error bars denote the standard deviation from three individual experiments. *P* value was calculated by Student’s T-test.

Gene expression (transcription) is generally regulated by TFs binding to specific sites. Therefore, changes of binding stability (*k*_*off*_) are expected to have a strong influence on gene expression level. To test this, we performed cell-based luciferase assays with GR and BMAL1-CLOCK interacting with smGRE and emE-box respectively, representing two opposite transcriptional regulation modes of DNA methylation (Fig. 2 and Supplementary Fig. S6).

In this cell-based luciferase assay, full length GR and BMAL1-CLOCK were overexpressed in HEK-293T cells and used as activators. The results showed that the relative luciferase activity, which was activated by GR, increased from 155 ± 8% to 494 ± 20% when the binding site GRE was replaced with the methylated type smGRE (Fig. 2). The luciferase activity was normalized by the control cells without GR over-expression (Fig. 2). Also as a control, the same luciferase assays were performed in the GR over-expressed but without dexamethasone treated HEK-293T cells. Very little decrease (but with no statistical significance, T-test *P* = 0.45) of gene expression in smGRE-cells (GRE: 67 ± 4%; smGRE: 64 ± 5%) indicated that the methylation of GRE itself cannot increase the gene expression unless with the GR binding. Parallel assays showed the relative luciferase activity with the activation of BMAL1-CLOCK decreased from 164 ± 12% to 102 ± 10% when the binding site E-box was replaced with the methylated type emE-box (Fig. 2).

Both GR and BMAL1-CLOCK results demonstrated that the methylation of TF binding sites could influence the tested protein expression level (luciferase activity). However, the quantitative relationships between the gene expression and the binding stability (*k*_*off*_) or affinity (*K*_*D*_) were complex in these cell-based assays, because the protein concentrations were hard to be determined in cells. Another reason is that the full length proteins underwent allosteric effect during DNA-protein interaction. The allosteric effect of GR has been systematically studied several years ago by Yamamoto group^35^, showing that the gene expression level does not have monotonic correlation with the binding affinity of GR interacting with different binding sites.

### Crystal structures of methylated DNA – protein complexes

Three crystal structures, unmethylated GRE-GRDBD, methylated smGRE-GRDBD and mmGRE-GRDBD complexes were solved at a resolution of ~2.3 Å. To eliminate crystal-packing effects, all GREs used for crystallization are the same sequence, and all crystals have been crystallized in the same space group P2_1_2_1_2_1_ with similar unit cell parameters (Supplementary Table S1).

**Figure 3:**
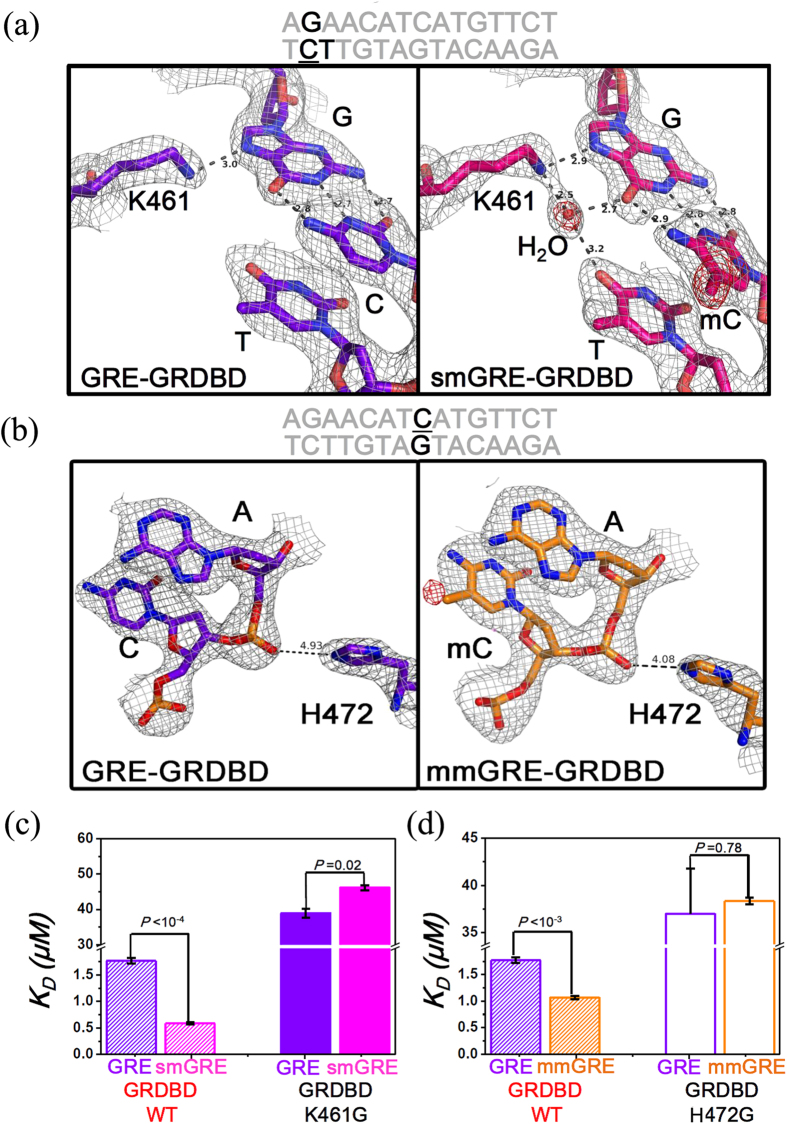
Structural studies of the methylated GRE-GRDBD complex. **(a,b)** The 2Fo-Fc electron density map (contoured at 1.0 σ) is shown in gray and the omit map (indicating the water molecule and methyl of mC, Fo-Fc map contoured at 3.0 σ) is shown in red. New hydrogen bonds between K461 of GRDBD and both mC-paired guanine and thymine (AGAACATGATGTTCT) were produced through a well-ordered water molecule when the cytosine was methylated in the smGRE-GRDBD complex. The electrostatic interaction between H472 of GRDBD and the phosphate group of adenine next to the mC (AGAACATCATGTTCT) is enhanced, with a significant distance decrease of ~0.85 Å when the cytosine was methylated in the mmGRE-GRDBD complex. **(c)** Dissociation constants *K*_D_ of mutant K461G of GRDBD binding to GRE or smGRE. **(d)** Dissociation constants *K*_D_ of mutant H472G of GRDBD binding to GRE or mmGRE. These affinities were all measured by ITC (Supplementary Figs S9 and S10), the final *K*_*D*_ is the average of two independent titrations and error bars denote the standard deviation. *P* value was calculated by Student’s T-test.

The smGRE-GRDBD and mmGRE-GRDBD structures show some significant features not seen in the previous complex structures. Firstly, the methyl groups of mCs within the GRE do not directly interact with the protein (Supplementary Fig. S7). Secondly, in the smGRE-GRDBD complex, K461 interacts with the mC-pairing guanine (AGAACATGATGTTCT) through two hydrogen bonds; one directly and the other through a bridging water molecule (Fig. 3a), this water molecule formed a hydrogen bond with thymine (AGAACATGATGTTCT) next to mC. However, in the GRE-GRDBD complex, K461 only forms one hydrogen bond with the guanine, this hydrogen bond can be found in all GRE-GRDBD complex crystal structures crystallized in the same space group P2_1_2_1_2_1 _35, and there are no water molecule to form hydrogen bonds in these crystal structures (Supplementary Fig. S8b), demonstrating that methylation of the GRE increases the number of hydrogen bonds between the DNA and protein (Fig. 3a). Similarly, more hydrogen bonds were also found on the other side of smGRE-GRDBD complex compared to GRE-GRDBD complex (AGAACATCATGTTCT. Supplementary Fig. S8a). The affinity of the mutant K461G of GRDBD binding to smGRE was not increased compared to its binding to GRE (Fig. 3c and Supplementary Fig. S9), indicating the residue K461 is important for the recognition of methylation.

Similar changes have also been observed in the mmGRE-GRDBD complex structure. The electrostatic interaction between H472 and the phosphate group of adenine next to the mC (AGAACATCATGTTCT) was enhanced, as evidenced by a significant distance decrease by ~0.85 Å between the oxygen atom of the phosphate group and the nitrogen atom of H472 when compared with the GRE-GRDBD complex (Fig. 3b). The affinities have no significant difference between the mutant H472G of GRDBD binding to GRE and mmGRE (Fig. 3d and Supplementary Fig. S10), indicating the residue H472 is important for the recognition of methylation as well.

Interestingly, H472 is an important residue positioned within the “lever arm” loop of GRDBD, proposed by Yamamoto group^35^, the “lever arm” is structurally sensitive to small environmental shifts and can modulate GR activity. We have compared our three high resolution crystal structures, and found that the conformations of the “lever arm” loops in smGRE-GRDBD and mmGRE-GRDBD complexes have indeed changed (Supplementary Fig. 11a), but the changes were smaller than that caused by the sequence mutation of GRE (Supplementary Fig. 11b)^35^. These observations suggested that the transcriptional activity of GR could be affected by methylation of cytosines in GRE, consistent with the results from the luciferase assay with GR.

**Figure 4:**
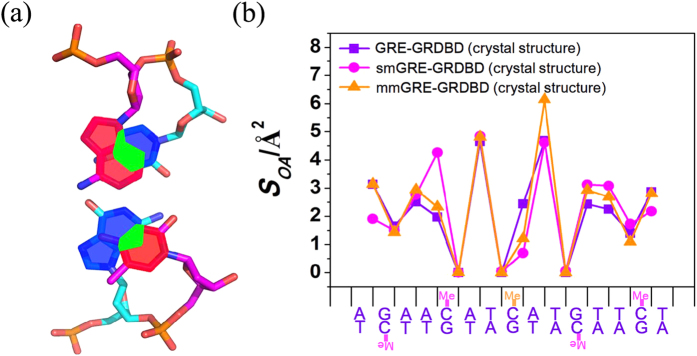
Overlapping area (*S*_*OA*_) analysis of base pair steps. **(a)** Definition of overlapping area (*S*_*OA*_), green region in the figure. **(b)** The *S*_*OA*_ of the GRE-GRDBD, smGRE-GRDBD and mmGRE-GRDBD complexes in the crystal structures were calculated and compared.

A major factor affecting DNA stability is the stacking interactions between base pair steps (defined as two adjacent base pairs from 5′ end to 3′ end, Supplementary Fig. 12), involving steric repulsions between exocyclic groups and π-π interactions between the heterocyclic ring of stacked base pairs^36^,^37^. Such effects can be evaluated by the value of overlapping area, *S*_OA_, between the heterocyclic rings of bases of two neighboring base pairs (the *S*_OA_ is defined at the plane perpendicular to the helix axis of DNA and the van der Waals radii of atoms are not considered, Fig. 4a). The *S*_*OA*_ of three GRE-GRDBD complexes (GRE-GRDBD, smGRE-GRDBD and mmGRE-GRDBD) in the crystal structures were calculated using *3DNA*^38^ and the comparison is shown in Fig. 4b. Interestingly, *S*_OA_ is very sensitive to the nature of the base steps and the most obvious change as a result of cytosine methylation was in the dinucleotide base step 5′-AC-3′ context, increasing in the *S*_OA_ values (Fig. 4b). There is no difference about protein/DNA interaction around 5′-AmC-3′compared to 5′-AC-3′, but the DNA structure was changed mostly at this context due to the methylation in the smGRE-GRDBD complex. The change of *S*_*OA*_ values also occurred in the mmGRE-GRDBD complex, but smaller than that of smGRE-GRDBD complex. All the information indicates that the methylation of cytosine in the GRE may influence the conformation of the DNA through a change of stacking interactions between the base pair steps and indirectly change the interaction with protein.

We then aligned these three complexes, the root mean square deviation (RMSD) between GRE and smGRE is 0.910 Å over all atoms, the RMSD between GRE and mmGRE is 0.352 Å over all atoms. Therefore, the conformational change of smGRE is larger than that of mmGRE, and the changes of *S*_*OA*_ in a way reflect their conformational changes.

### MD simulation of methylated DNA–protein complexes

We were not able to obtain crystals of a methylated E-box-BCDBD complex, probably because of the weakened protein/DNA interaction. To gain insight into the protein/DNA interaction for these complexes, molecular dynamics (MD) simulations were performed.

**Figure 5:**
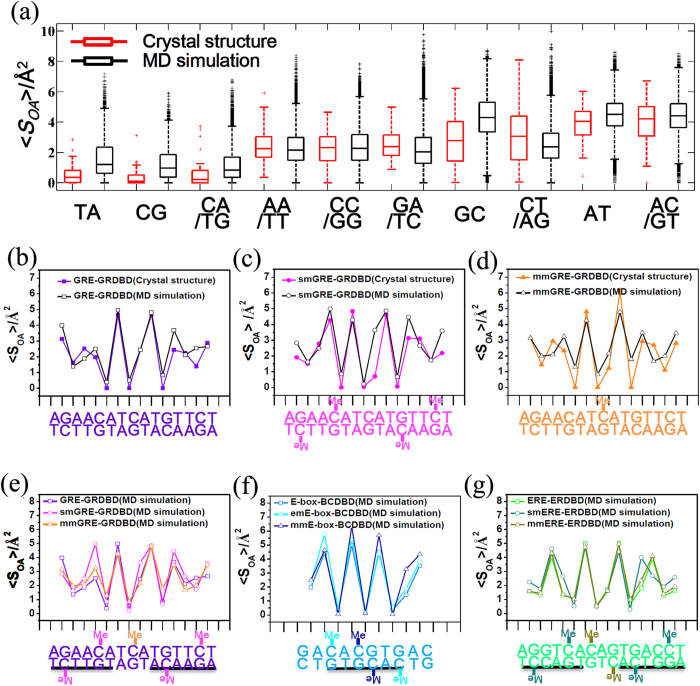
Comparison of the *S*_*OA*_ calculated from crystal structures and MD simulations, and the *S*_*OA*_ of DNA-protein complexes studied by MD simulations. **(a)** Comparison of the *S*_*OA*_ distribution of all dinucleotide base steps between 30 free B-form DNA crystal structures (red) from the PDB database (Table S2) and MD simulations (black) of free DNAs plotted as boxes. **(b–d)** Comparison of the average *S*_*OA*_ between the crystal structures and MD simulations of GRE-GRDBD, smGRE-GRDBD and mmGRE-GRDBD complexes. **(e–g)** Comparison of the average *S*_*OA*_ of each dinucleotide base steps in **(e)** GRE-GRDBD, smGRE-GRDBD and mmGRE-GRDBD complexes, (**f**) E-box-BCDBD, emE-box-BCDBD and mmE-box-BCDBD complexes, **(g)** ERE-ERDBD, smERE-ERDBD and mmERE-ERDBD complexes. Black underlines indicate the direct binding region.

We first tested whether MD simulations can provide useful information on DNA structures. Simulations were performed on free DNAs of different sequences and the *S*_OA_ of each dinucleotide base step were calculated using *3DNA*^38^ from the simulation trajectories. The *S*_OA_ of all dinucleotide base steps of 30 high-resolution free B-form DNA structures from the PDB database (Supplementary Table S2) were also calculated. The distributions of all ten different dinucleotide base steps were drawn using the box plot and compared with the MD simulations (Fig. 5a). The distributions of *S*_OA_ of dinucleotide base steps obtained from MD simulations and the crystal structures are in reasonable good agreement, supporting the idea to apply MD simulations for *S*_OA_ calculations.

Next, MD simulations were performed for the GRE-GRDBD, smGRE-GRDBD and mmGRE-GRDBD complexes at 300 K, and the averaged *S*_OA_ of each dinucleotide base step were calculated from the simulation trajectories. The *S*_OA_ of the three complexes from MD simulations and crystal structures were found to agree quite well (Fig. 5b–d).

In the GRE-GRDBD complexes, the methylation of cytosine in GRE changes the DNA conformation as seen from the increase of the *S*_OA_ of the 5′-AC-3′ context (Fig. 5e). Furthermore, the *S*_OA_ increase of 5′-AC-3′ in the smGRE-GRDBD complex is larger than the *S*_OA_ increase in the mmGRE-GRDBD complex, agreeing with the previous experimental data.

**Figure 6:**
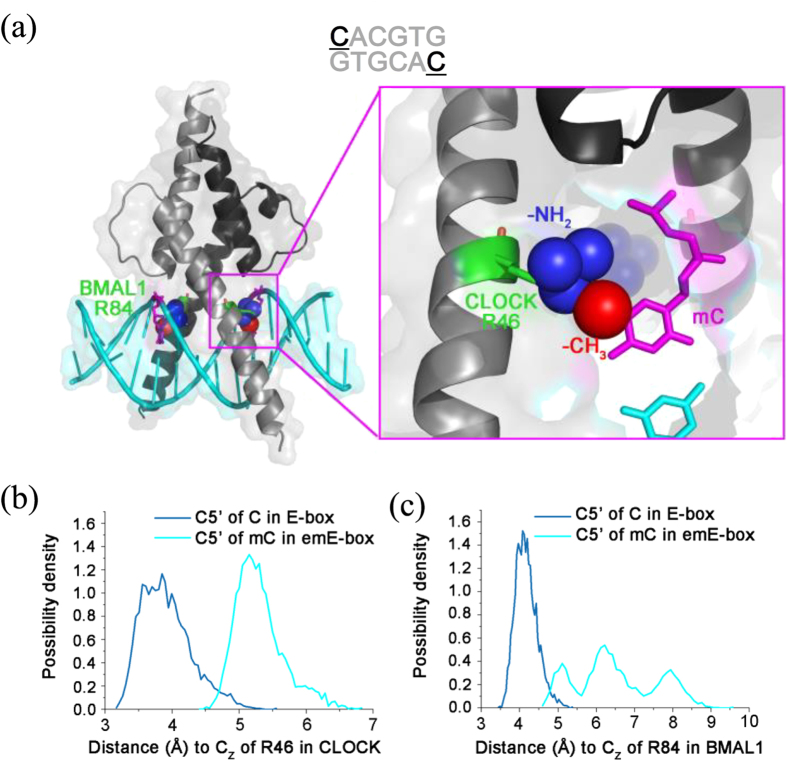
Exclusion effect in smE-box-BCDBD complex studied by MD simulations. **(a)** The mutated structure (the cytosines were directly mutated into methylated cytosines by Coot) of the emE-box-BCDBD complex from PDB ID: 4H10. Green residues represent R84 of BMAL1 and R46 of CLOCK. Blue spheres represent their terminal amino groups. Mutated cytosines are shown as magenta cartoon and the red sphere represents its methyl group. The existence of the methyl group may repel proteins to reduce DNA binding affinity. (**b**) The distribution of the distance between the carbon atom C_Z_ of CLOCK R46 and the carbon atom C5 of the cytosine (CACGTG) in Ebox and emEbox. **(c)** The distribution of the distance between the carbon atom C_Z_ of BMAL1 R84 and the carbon atom C5 of the cytosine (CACGTG) in Ebox and emEbox. Data in (**b,c)** come from MD simulations.

We further used MD simulations to study the E-box-BCDBD, emE-box-BCDBD and mmE-box-BCDBD complexes (Fig. 5f). The *S*_OA_ in the 5′-AC-3′ context changes after the cytosines in the E-box are methylated, indicating a change in the conformation of the DNA because of cytosine methylation. Furthermore, close inspection of the calculated structures shows clear differences between the emE-box-BCDBD and mmE-box-BCDBD complexes. In the emE-box-BCDBD complex, the BCDBD interacts directly with the two methylated cytosines (Fig. 6a). The hydrophobic methyl group of mC may exclude BCDBD at R46 in CLOCK and R84 in BMAL1, because of the hydrophilic nature of R46 and R84 (Fig. 6a). The calculation for the emE-box-BCDBD complex showed that the C5 carbon of the cytosine (position of the methyl group) stays more than 2~5 Å away from the amino group of R46 or R84, as expected (Fig. 6b,c). A quite different scenario is observed for mmE-box-BCDBD: the methyl groups of the middle methylated cytosines are situated in a hydrophobic environment (Supplementary Fig. S13a), and the methylation of these cytosines does not affect the BCDBD/E-box interaction significantly (Supplementary Fig. S13b,c). These differences in protein/DNA interactions partly account for the decreased stabilities of emE-box-BCDBD and mmE-box-BCDBD, compared with E-box-BCDBD.

In contrast, the *S*_OA_ of ERE-ERDBD, smERE-ERDBD and mmERE-ERDBD complexes have smaller differences (Fig. 5g) compared with the cases of GRE-GRDBD and E-box-BCDBD (Fig. 5e,f), indicating that the DNA conformation remains largely unchanged by the methylation of the cytosine. Interestingly, the affinity between ERE and ERDBD is also insensitive to methylation.

### BisChIP-seq revealed that GR can bind to the highly methylated sites but BMAL1-CLOCK can not

The common view concerning gene regulation by CpG methylation is that the TFs are repressed by binding of MBPs^18^. Although the binding ability of MBPs to non-CpG has been identified^19^,^20^, they only suggested that non-CpG methylation enhances the binding of MBPs in the gene body, the role of non-CpG methylation in the promoter region remains unclear^21^.

**Figure 7:**
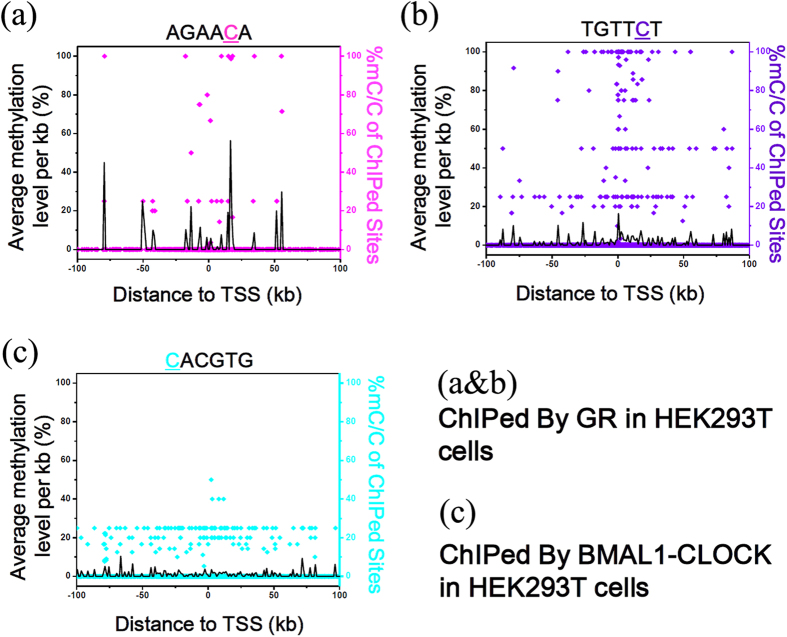
BisChIP-seq in HEK-293T cells revealed GR can bind to the highly methylated sites but BMAL1-CLOCK not. **(a,b)** The methylation level of individual cytosine in “AGAACA” or “TGTTCT” (the “AGAACA” sequence represents half of the GRE, which is a strong candidate for GR binding^35^), and their distance to the nearest transcriptional start site (TSS) in the ChIPed DNA from HEK-293T cells. (**c**) The methylation level of individual cytosine in “CACGTG” (the typical E-box binding site of the BMAL1-CLOCK complex^34^), and their distance to the nearest transcriptional start site (TSS) in the ChIPed DNA from HEK-293T cells. The black line is the average methylation level for every 1 kb, which indicates the ratio of the hypermethylation sites.

To verify if TFs can bind to the highly methylated sites on the chromatin of cells, we performed bisulfite sequencing of chromatin immunoprecipitated DNA (BisChIP-seq) for GR and BMAL1-CLOCK in HEK-293T cells. The methylation level of the fifth cytosine in “AGAACA” or “TGTTCT” sequence in the DNA immunoprecipitated by GR, and their distance to the nearest transcriptional start site (TSS) were shown in Fig. 7a,b. In the promoter region close to the TSS, GR can bind to both the unmethylated sites and highly methylated sites on the chromatin of cells. It means GR most likely can not be blocked by MBPs in the promoter region, due to its increased binding affinity to methylated sites.

However, the methylation level of the first cytosine in “CACGTG” sequence in the DNA immunoprecipitated by BMAL1-CLOCK (Fig. 7c) shows very different profile compared with GR. All the binding sites immunoprecipitated by BMAL1-CLOCK within the promoter region close to the TSS are hypo-methylated, suggesting that BMAL1-CLOCK can only bind to the hypo-methylated sites in cells. The possible reason is the methylation of “CACGTG” decreases the binding affinity with BMAL1-CLOCK.

### Methylation level of GRE and E-box are changed during brain development

According to the previous published methylome sequencing data of adult mammalian brains^10^, we then investigated the GR, BMAL1-CLOCK and ER binding sites (GRE, E-box and ERE) within the 35-day old human middle frontal gyrus (tissue), 53-year old female and 55-year old male human dorsal prefrontal cortices (neuron). The methylation levels of cytosine within “AGAACA”, “CACGTG”, and “AGGTCA” sequences were altered during brain development. For a portion of these sequences, the methylation level is significantly higher in aged brain cells than in the young ones (Supplementary Fig. S1). According to the above experiments, we hypothesize that the variation of methylation of these potential GR and BMAL1-CLOCK binding sites could regulate GR and BMAL1-CLOCK related genes at different statuses during brain development, but the variation of methylation of potential ER binding sites cannot.

## Discussion

We have in this report, proposed a direct and simple mechanism for transcriptional regulation by DNA methylation, we have shown in our work that altered affinity/stability between TFs and DNA elements caused by DNA methylation (particularly by non-CpG methylation) can serve as a direct source for fine tuning of gene expression. However, gene regulation in reality could be much more complex, previous research has mainly focused on gene repression by DNA methylation (particularly for CpG methylation). The general gene expression could be regulated by at least three aspects by DNA methylation. Firstly, the binding of TFs could be blocked by MBPs at promoter regions due to methylation; secondly, when TF has a chance to bind to its target, the binding stability could be modified by direct interacting with the methylated DNA sequences; thirdly, after a gene transcription had been initiated, the elongation could be blocked by MBPs bound on gene body due to DNA methylation.

GR is expressed in most cells and regulates the transcription of thousands of genes that affect many life processes^39^. For example, it is abundantly expressed in the brain, in both neuronal and non-neuronal cells^40^,^41^,^42^,^43^. In neuronal cells, GR targeted sites containing GRE are more often located in the vicinity of genes involved in general cellular functions^43^, and GR regulates a large number of genes through direct binding^44^. Therefore, the increase of the methylation level of GREs in the brain can regulate many genes through an increase in GR binding.

The heterodimeric BMAL1-CLOCK complex controls the expression of a large number of genes related to the circadian rhythm^45^. It is thus reasonable to speculate that the increase of the methylation level in the brain during development could impact on the circadian rhythm through reduction of BMAL1-CLOCK binding and change the behavior of mammals, e.g., the wake and rest period for infants may be quite different to that of older people.

The most important role of DNA is to encode genes that will be transcribed from DNA to RNA, and this process is regulated by DNA/RNA binding proteins. All genes can be switched between “on” and “off” states during cell metabolism and development. In the past, the actions of genes were mostly considered to be in either “on” or “off” states. Recently, however, some fine tuning mechanisms^46^ for the gene switch have been studied and defined, such as allostery through DNA^31^ and conformational changes of nucleosomal DNA^47^. These effects are generally on the order of 2–3 fold in magnitudes, well in the range of recessive inheritance, where only one functional copy of the parental genes is expressed. Here, we would like to propose another fine-tuning mechanism for gene regulation, namely through non-CpG methylated DNA, which also affects DNA/protein binding affinity by 2–3-fold. The current study also demonstrates that different proteins undergo varied regulation and control when their DNA binding sites are methylated. The current viewpoint that repression is the sole outcome of DNA methylation should be reconsidered. Thus, the effect of DNA methylation should be analyzed on a case-by-case basis for a particular TF with respect to the regulation of gene expression. There are several possibilities for general TFs: (i) TFs prefer to bind to methylated DNA due to enhanced binding affinity; (ii) TFs prefer to bind to unmethylated DNA because of reduced binding affinity with 5mC modification; (iii) the TFs have no preference towards methylation of DNA; (iv) for one particular TF and its binding site, when the binding sequence/site is changed by methylation, the effect may be varied, e.g. for GR, besides preferring to bound to some methylated DNA sequences, it does not prefer a binding site with a methylated CpG^30^, this phenomenon has also been observed in the nucleosome positioning on methylated DNA^27^,^28^,^29^.

Finally, with an enriched knowledge and understanding of the mechanisms of DNA methylation and allosteric effects, it is clear that DNA binding control is not merely an ON/OFF switch, but also performs general fine-tuning with different mechanistic details. These detailed considerations will guide us to carry out more thorough analyses with promoter regions^48^,^49^ and with ChIP-seq data.

## Methods

### The k_off_ and K_D_ measurements

The dissociation rates *k*_*off*_ between proteins and DNA were measured by a single molecule assay, following our previous publication (Jin *et al*.^47^; Kim *et al*.^31^). The dissociation constants *K*_*D*_ between proteins and DNA were measured by ITC (Isothermal Titration Calorimetry) using the ITC200 (GE Healthcare) at 25 °C. A more detailed description about these two measurements can be found in the Supplementary Methods.

### Cell-based luciferase assay

Both the GR and BMAL1-CLOCK luciferase assays were performed following the previous literatures^34^,^35^, except we did them using the methylated plasmids. GRE, smGRE, E-box and emE-box were cloned into pGL3-promoter vector (Promega). The pGL3-promoter vector contains TATA box after XhoI site in a SV40 promoter which drives a luciferase reporter gene (firefly luciferase). Details can be found in the Supplementary Methods.

### Crystallization and structure determination

The GRE-GRDBD, smGRE-GRDBD and mmGRE-GRDBD complexes were mixed with equal volume of reservoir at 18 °C and the crystals were grown by hanging drop method respectively. GRE-GRDBD crystals grew in 50 mM Na Cacodylate, pH 6.5, 2.25 mM Spermine, 18 mM MgCl_2_, 9% isopropanol; mmGRE-GRDBD crystals grew in 50 mM Tris, pH 7.5, 200 mM KCl, 50 mM MgCl_2_, 10% PEG4000; smGRE-GRDBD crystals grew in 50 mM HEPES, pH 7.0, 100 mM KCl, 10 mM MgCl_2_, 5% PEG400. Crystals were then soaked in crystallization solutions with 35% ethylene glycol for several minutes before flash frozen in liquid nitrogen. The diffraction data were collected on the KEK Photon Factory, the complex structures were determined by Molecular replacement methods and refined by PHINEX software package. A more detailed description about the data collection and structure determination can be found in the Supplementary Methods.

### MD simulation

All three complexes (GRE-GRDBD, E-box-BCDBD and ERE-ERDBD) were simulated using AMBER11^50^. The initial structures of GRE-GRDBD, smGRE-GRDBD, mmGRE-GRDBD, E-box-BCDBD and ERE-ERDBD complexes were obtained from their crystal structures, the initial structures of emE-box-BCDBD, mmE-box-BCDBD, emERE-ERDBD and mmERE-ERDBD were obtained by single mutation using Coot^51^ from their unmethylated crystal structures, and the initial structures of naked DNA were the nucleic acid part of corresponding complex.

Each of the complexes was immersed into a cubic (for naked DNA) or a truncated octahedral (for DNA-protein complex) box with a 12 Å water described by SPC/E model^52^ in each direction. The sodium ions were added into boxes as counter ions.

The systems were first minimized through 500 steps of steepest descent minimization and a following 500 steps of conjugate gradient minimization. They were then minimized using 1000 steps of steepest descent and 1500 steps of conjugate gradient minimization. After that the system was heated to 360 K for further relaxation and equilibrated at the temperature for 200 ps before they were cooled down to 300 K.

Finally, 50 ns production runs were performed at 300 K using the Langevin dynamics with a friction coefficient of 5ps^−1^. The pressure was adjusted to 1 atm by Berendsen weak-coupling algorithm^53^ with a relaxation time constant of 2.0 ps. All dynamic runs used an integral time step of 2 fs. More details can be found in the Supplementary Methods.

### ChIP-Bisulfite sequencing (BisChIP-seq)

For GR, GR-pCMV14 and DNMT3A-pLX304 (because the non-CpG methylation in somatic cells are always low, and the expression of DNMT3A in HEK293T cell is also very low, so we added this vector to increase the non-CpG methylation of HEK293T cell’s chromatin.) vectors were co-transfected into HEK-293T cells using the Genscort II transfection reagent (Wisegen), and Dexamethasone (Sigma Aldrich) was added to a final concentration of 4 nM after 24 hours to make GR transfer into the nucleus. For BAML1-CLOCK, BMAL1-pCMV14, CLOCK-pCMV14 and DNMT3A-pLX304 vectors were also co-transfected into HEK-293T cells but without dexamethasone added. Then cells were collected after 24 hours incubation followed by the ChIP experiment which was performed as described in previous studies^54^,^55^,^56^.

Cells were cross-linked by 1% formaldehyde for 8 minutes at room temperature and the glycine was added to stop the reaction at a final concentration of 0.125 M. Cross-linked cells were then washed by PBS (phosphate buffer saline) several times, before resuspended in the lysis buffer (1% SDS, 50 mM Tris-HCl pH 8.0, 10 mM EDTA) on ice for 5 minutes. The lysate was aspirated into Covaris tube and was sonicated by Covaris S2 (Covaris) with a scheduled program (Duty Cycle: 5%, Intensity: 4; Cycles: 200; Time: 60 s; Total time: 6 minutes). Afterwards, the lysate was diluted in RIPA buffer (0.5 mM EGTA, 140 mM NaCl, 10 mM Tris-HCl pH 7.5, 1% Triton-X100, 0.1% SDS, 1% EDTA) and then mixed overnight with Dynabeads Protein G (Invitrogen) for immunoprecipitation which were sealed with Anti-Flag antibody (Sigma) at 4 °C (we also did a control experiment with antibody IgG (Sigma) to evaluate the immunoprecipitation step). After that the beads were ligated with target proteins and DNA was washed with RIPA buffer several times. Then the beads were resuspended in Complete Elution buffer (1% SDS, 20 mM Tris-HCl pH 7.5, 200 ug/ml proteinase K, 5 mM EDTA, 50 mM NaCl) at the temperature of 68 °C for 2 hours. After the digestion of proteinase K, beads were removed by a magnetic shelf, and samples were mixed with the same volume of phenol-chloroform-isoamyalcohol (25:24:1) (Sigma) to extract DNA. After centrifugation, the supernatant was aspirated and mixed with the same volume of chloroform-isoamyalcohol (24:1) (Amresco) to remove phenol and proteins left through centrifugation. Next, 100% cold ethanol and LPA was used to mix with samples, incubate at −80 °C for at least one hour to make DNA precipitate. Discard the supernatant after centrifugation and the pellet is the expected DNA. The pellet was then washed with 70% cold ethanol, remaining ethanol volatilized at room temperature. Generally we can acquire 50 ng DNA from about 10^6^ cells with GR or BAML1-CLOCK.

Libraries were constructed with NEBNext DNA Library Prep Master Mix Set for Illumina (NEB). After end repair and dA-tailing, methylated adaptors (NEBNext Multiplex Oligos for Illumina) were applied to ligate with DNA followed by bisulfite conversion using EZ DNA Methylation Kit (Zymo research). We also did a control experiment without the bisulfite conversion step, which was a normal ChIP-seq assay, to evaluate the significance of the ChIP-seq signal (a typical result of the BisChIP-seq, ChIP-seq and non-specific antibody ChIPed signal within a same region were presented in Supplementary Fig. S14, which is for example. Both the peak range and width indicated that the BisChIP-seq assay has worked well.). Then DNA was recovered using AMPure XP beads (Beckman Coulter) after PCR amplification and sequenced by Hiseq2000 (Illumina). Sequencing data were processed using Bismark^57^ and programs written by Perl. Details can be found in the Supplemental Experimental Procedures.

## Additional Information

**Accession codes:** The following coordinates have been deposited in the RCSB Protein Data Bank. The GRE-GRDBD is the GRDBD and GRE complex (PDB ID: 5EMQ), smGRE-GRDBD is the GRDBD and smGRE complex (PDB ID: 5EMC), mmGRE-GRDBD is the GRDBD and mmGRE complex (PDB ID: 5EMP). BisChIP-seq data can be downloaded from the National Center for Biotechnology Information GEO (GSE64171).

**How to cite this article**: Jin, J. *et al*. The effects of cytosine methylation on general transcription factors. *Sci. Rep.* **6**, 29119; doi: 10.1038/srep29119 (2016).

## Supplementary Material

Supplementary Information
