# Supplementary material for: The effects of cytosine methylation on general transcription factors
Source: Sci Rep. 2016 Jul 7;6:29119. doi: 10.1038/srep29119 (PMC4935894; doi:10.1038/srep29119)
Supplement: Supplementary Information [file srep29119-s1.pdf]

**Supplementary Information**

# The effects of cytosine methylation on general transcription factors

Jianshi Jin, Tengfei Lian, Chan Gu, Kai Yu, Yi Qin Gao, Xiao-Dong Su

## Supplementary Methods

### 1. Protein expression and purification

The preparation of GRDBD and ERDBD has been described previously[1], and the preparation of GRDBD mutants K461G and H472G were similar to the native GRDBD. GRDBD and ERDBD were constructed into the pET28a vector and overexpressed in *Escherichia coli* BL21 (DE3) strains, and protein expression was induced by the addition of isopropyl β-D-1-thiogalactopyranoside (IPTG) to a final concentration of 0.5 mM. Cells were left to grow overnight at 18 ℃. Bacteria were then collected and resuspended in buffer A (20 mM Tris-HCl, pH 7.5, 1 M NaCl) before sonication and centrifugation. Subsequently, the supernatant was loaded onto a Ni-chelating column (GE Healthcare, PA, USA) and target proteins were eluted by imidazole at a concentration of 100–200 mM. However, for GRDBD crystallization, the overexpressed protein was further digested by thrombin (GE Healthcare) at 18 ℃ overnight to remove the His-tag, followed by gel filtration chromatography (Superdex 75, GE Healthcare) for final purification in buffer B (20 mM Tris-HCl, pH 7.5, 100 mM NaCl and 5 mM DTT). Cloning and expression of Bmal1 and Clock bHLH domains were also described in previous work[2]. Both proteins were subcloned into pET21b and overexpressed in *E. coli* BL21(DE3). After the bacteria were induced at 18 ℃ overnight with IPTG, harvested bacteria containing the Bmal1 and Clock bHLH domains were

resuspended in buffer C (20 mM Tris-HCl, pH 7.8, 500 mM NaCl) before sonication and centrifugation. Both proteins were purified by $Ni^+$ column (HiTrap $Ni^+$ chelating column, GE Healthcare) and eluted by imidazole at a concentration of 100–300 mM. The two proteins were then mixed as equal molar amounts followed by gel filtration chromatography (Superdex 75, GE Healthcare) in buffer D (20 mM Tris-HCl, pH 7.8, 200 mM NaCl and 1 mM DTT) to produce BCDBD.

## 2. Protein labeling

Purified proteins for the single molecule assay were labeled by reaction with Cy3B–maleimide (GE Healthcare) at the surface cysteine for GRDBD and BCDBD, and the C-terminal engineered cysteine for ERDBD. Protein labeling was performed according to the Amersham Cy™3B mono-reactive dye protocol (GE Life Sciences) and proteins were purified with a gel filtration desalting column (PD MinTrap G-25, GE Life Sciences), according to the product manual, which have been described previously[1].

## 3. Single molecule assay

The single molecule assay which was used to measure the dissociation constants, $k_{\mathrm{off}}$, between proteins and DNA were performed following the protocol in previous publications[1,3]. Briefly, the biotinylated DNA (Supplementary Table S3) used in the single molecule assay were synthesized by Sangon Biotech (Shanghai, China) and annealed to dsDNA respectively. We then immobilized the biotinylated dsDNA onto a coverslip within a homemade flow-cell via the biotin-streptavidin linkage, incubating

with the biotinylated dsDNA at a concentration of 50 pM in buffer 1 (50 mM Tris-HCl, pH 8.0, 10 mM NaCl, 0.1 mM $Na_2$EDTA and 1 mg/ml BSA). After removing the extra biotinylated dsDNA in the flow-cell with buffer 1 washing, we injected the labeled proteins (GRDBD, BCDBD or ERDBD) at a concentration of 10 nM in imaging buffer (20 mM Tris, pH 7.8, 100 mM NaCl, 500 g/mL BSA (Shanghai Excell Biology. Inc., Shanghai, China), 10% (v/v) glycerol, 0.8% (w/v) glucose, 1 mg/mL glucose oxidase, 0.1 mg/mL catalase and 2 mM trolox (all reagents were purchased from Sigma-Aldrich, MO, USA, if not otherwise noted)) into the flow-cell for pre-binding. The unbound proteins were removed by washing immediately before data collection. During the acquisition of images, the flow rate of the imaging buffer was set to ~1 mm/s using a syringe pump (Model LSP02-1B, Longer Pump, Hebei, China).

The single molecule fluorescence imaging was carried out on an inverted microscope (Nikon ECLIPSE Ti-E, Nikon Instruments Inc. Tokyo, Japan) with total internal reflection (TIR) illumination. An area consisting of approximately $10^{-4}$ $cm^2$ was illuminated with ~5 $W/cm^2$ by focusing the beam of a solid state 532 green laser (MGL-III-532/100mM, Changchun New Industries Optoelectronics Tech. Co., Ltd, Changchun, China) into the back aperture of a 100× objective lens (TIRF, 1.49, PlanApo, oil, Nikon Instruments Inc.). Fluorescence measurements were collected using the same objective lens through a dichroic beam splitter (model Di01-R532-25*36, Semrock, NY, USA) and a long pass filter (model BLP01-532R-25, Semrock) to be imaged on a back-illuminated electron multiplying charge coupled device (EMCCD) camera (iXon Uitra 897, Andor Technology, Belfast, UK). A fluorescent

image (100 × 100 $\mu m^2$) was then recorded every 0.3 s for a duration of 5–10 min, containing hundreds of fluorophores (Supplementary Fig. S3a: before protein dissociation; Supplementary Fig. S3b: after protein dissociation. These images were done with GRDBD, which is for example).

Images were then processed by algorithms written in Matlab (The Mathworks, Inc., Natick, MA), as described previously[1,3]. Figures of $k_{off}$ were performed by Origin.

The control experiments of non-specific binding to surface were performed for all the three TFs respectively. Here we only showed the images of GRDBD for example. The results showed that only few of the TFs will bind to the surface (Supplementary Fig. S3c) or DNA without specific binding site (Supplementary Fig. S3d). These control experiments demonstrated that in the single molecule dissociation assay, most of the bright spots were the TFs bound on their specific binding sites within the immobilized DNA, the non-specific binding to both surface and DNA can be ignored.

Other control experiments, such as the labeling effect and laser power effect for the protein binding et. al., have been done in our previous works [1,3], because the labeled proteins GRDBD and ERDBD were both used for other single molecule experiments in those works.

## 4. ITC assay

Normal DNA and methylated DNA (Supplementary Table S4) used for the isothermal titration calorimetry (ITC) assay were also purchased from Sangon Biotech. All the GREs and E-boxes were dissolved in buffer 2 (20 mM Tris-HCl, pH 7.5, 100 mM NaCl, 5 mM DTT and 1 mM $Na_2$EDTA) and the EREs in buffer 3 (10 mM MES, pH 6.0, 100

mM NaCl, 5 mM DTT, 1 mM $Na_2EDTA$), which are the same buffers as the corresponding proteins, and then gel filtration chromatography (Superdex 75, GE Healthcare) was used for further purification. All proteins were titrated into the DNA solution using an ITC200 (GE Healthcare) at 25 ℃. To determine affinities between proteins and DNA: 0.2 mM GRDBD was titrated into 0.017 mM GREs, 0.2 mM ERDBD was titrated into 0.017 mM EREs, and 0.4 mM BCDBD was titrated into 0.036 mM E-boxes. Each experiment was repeated three times. All samples have their control experiments respectively, which the proteins (0.2 mM GRDBD, 0.2 mM ERDBD, and 0.4 mM BCDBD) were titrated into the buffers only (buffer 2 for GRDBD and BCDBD, and buffer 3 for ERDBD). And these controls (Supplementary Fig. S5) were also used as background deduction during data processing. The thermograms were integrated by the Origin software and fitted to a single site binding model, and the error bar is the standard deviation of three repeats.

As control experiments, the affinities between GRDBD mutants and GREs were also measured by ITC. In the titrations of mutant K461G of GRDBD to GRE and smGRE: 0.2 mM mutant K461G of GRDBD was titrated into 0.017 mM GRE or smGRE respectively (Supplementary Fig. S9). In the titrations of mutant H472G to GRE and mmGRE: 0.2 mM mutant H472G of GRDBD was titrated into 0.017 mM GRE or mmGRE respectively (Supplementary Fig. S10). All these titrations were performed at 25 ℃, each of them was repeated two times, and the error bar is the standard deviation of two repeats.

## 5. Cell-based luciferase assay

Both GR and BMAL1-CLOCK luciferase assays were performed following the previous literatures [2,4], except we did them using the methylated plasmids. GRE, smGRE, E-box and emE-box were cloned into pGL3-promoter vector (Promega) between KpnI and XhoI sites by PCR and cyclization using two 5’ phosphorylated primers (which have overlapped sequence in the middle, Supplementary Table S5) respectively (Supplementary Fig. S6). The pGL3-promoter vector contains TATA box after XhoI site in a SV40 promoter which drives a luciferase reporter gene (firefly luciferase). All the DNAs generated in each step were purified by agarose gel and Zymoclean™ Gel DNA Recovery Kit (ZYMO Research). The T7 DNA ligase (NEB) was used to ligate the end to end paired DNA to form circular plasmids (pGL3-GRE, pGL3-smGRE, pGL3-E-box and pGL3-emE-box).

For GR luciferase assays, 20ng pRL-TK, 20ng pGL3-GRE (or pGL3-smGRE), 50ng pCMV14-GR and 50ng pCMV14 empty vector were transfected into cultured HEK293T cells at 60% confluence in 24-well plate by Genscort II transfection reagent. Then dexamethasone was added to a final concentration of 4 nM after 20 h to facilitate the transfer of GR into nucleus. Two non-activated controls were done for eliminate the effects by methylated DNA itself. The first was without GR expressed for both systems, respectively (20ng pRL-TK, 20ng pGL3-GRE (or pGL3-smGRE), 100ng pCMV14), then dexamethasone was added as before. The second was with GR expressed (20ng pRL-TK, 20ng pGL3-GRE (or pGL3-smGRE), 50ng pCMV14-GR and 50ng pCMV14 empty vector), but without dexamethasone treatment.

For BMAL1-CLOCK luciferase assays, 5ng pRL-TK, 20ng pGL3-E-box (or pGL3-emE-box), 50ng pcDNA3.1-Bmal1, 150ng pcDNA3.1-Clock and 200ng pcDNA3.1 empty vector were transfected into HEK293T cells at 60% confluence but without dexamethasone added. Co-transfection of 5ng pRL-TK, 20ng pGL3-E-box (or pGL3-emE-box), 400ng pcDNA3.1 without BMAL1-CLOCK expressed were done as control (the transfer of BMAL1-CLOCK into the nucleus is not controlled by any small molecule, so the other control experiment like GR has not done).

Cells were lysed with 1× Passive Lysis Buffer (Promega) after 24 h of incubation. The lysates were transferred into 1.5mL EP tubes for luciferase assays, which were performed using a Dual-Luciferase Reporter Assay System (Promega) according to the manufacturer's protocol. Luciferase activities were detected by GloMax 20/20 Single Tube Luminometer (Promega).

To eliminate the difference of the transfection efficiency in each individual experiment, we used an internal reference to normalize relative luciferase activity of each experiment. The internal reference was reported by another luciferase reporter gene (Renilla luciferase) which was co-transfected by a control plasmid (pRL-TK) in each individual experiment. The activity of each non-activated system without protein expressed was set as 100%, and the relative luciferase activity of each system were presented by percentage of their non-activated control.

## 6. Crystallization and structure determination

Normal DNA and methylated DNA (Supplementary Table S6) for crystallization were

purchased from Sangon Biotech and dissolved in buffer 4 (20 mM Tris-HCl, pH 7.5, 100 mM NaCl and 5 mM DTT). All DNA samples were heated to 95 ℃ and annealed slowly to 4 ℃ with further purification by gel filtration chromatography (Superdex 75, GE Healthcare) to remove single-stranded DNA.

GRDBD (0.5 mM) was mixed with three types of GREs in a 1:1 molar ratio and incubated on ice for 30 min. Then, the complexes were mixed with equal volume of reservoir at 18 ℃ and crystals were grown by the hanging drop method. GRE-GRDBD crystals grew in 50 mM Na-cacodylate, pH 6.5, 2.25 mM spermine, 18 mM $MgCl_2$, 9% isopropanol, mmGRE-GRDBD crystals grew in 50 mM Tris, pH 7.5, 200 mM KCl, 50 mM $MgCl_2$, 10% PEG4000, and smGRE-GRDBD crystals grew in 50 mM HEPES, pH 7.0, 100 mM KCl, 10 mM $MgCl_2$, 5% PEG400. Crystals were then soaked in crystallization solutions with 35% ethylene glycol for several minutes before flash freezing in liquid nitrogen.

Datasets of GRE-GRDBD crystals and mmGRE-GRDBD crystals were collected at KEK, Photon factory beamline BL1A, Tsukuba, Japan, using a Pilatus 2M CCD detector (Dectris). Datasets of smGRE-GRDBD crystals were collected at KEK, Photon factory beamline BL17A using a Quantum 270 CCD detector (Area Detector Systems Corporation). All the datasets were processed with the XDS [5] program and structures were determined by molecular replacement in Molrep [6] with the model 1R4R (PDB ID) using the CCP4 suite [7]. Structure models were improved by Coot [8] and refined by phenix.refine [9]. Final models were analyzed by Molprobity [10]. There are 94.9% of the residues of the GRE-GRDBD model in the favored region and 5.1% residues in the

allowed region with no outliers. The model of smGRE-GRDBD has 94.2% residues in the favored region and 5.1% residues in the allowed region and 0.7% residues as outliers. For the mmGRE-GRDBD model, the favored region contains 96.4% residues, the allowed region contains 2.9% allowed residues, and 0.7% of the residues are outliers. Crystallographic parameters of these models are listed in Supplementary Table S1. All structure figures were generated by PyMOL (Delano Scientific).

**7. MD simulation**

All three complexes (GRE-GRDBD, E-box-BCDBD and ERE-ERDBD) were simulated using AMBER11[11]. The initial structures of GRE-GRDBD, smGRE-GRDBD, mmGRE-GRDBD, E-box-BCDBD and ERE-ERDBD complexes were obtained from their crystal structures. The initial structures of emE-box-BCDBD, mmE-box-BCDBD, emERE-ERDBD and mmERE-ERDBD were obtained by single mutations using Coot[8] from their unmethylated crystal structures, and the initial structures of naked DNA were the nucleic acid part of the corresponding complexes. Naked DNA "5′-CG(CG)$_{13}$CG-3′" was also simulated for the 5′-CG-3′ context.

Each of the complexes was immersed in a cube (for naked DNA) or a truncated octahedral (for the DNA-protein complexes) box with a 12 Å water shell described by the SPC/E model[12] in each direction. Sodium ions were added into boxes as counter ions. Both the protein and nucleic acid parameters were taken from the AMBER ff10 parameter set[13] and the force field of 5-methylcytosine was obtained from a previous work[14]. For each system, the simulation procedure included energy minimization,

heating up and a production equilibrium simulation using the NPT ensemble.

The systems were first minimized through 500 steps of steepest descent minimization and a following 500 steps of conjugate gradient minimization. Then, the systems were minimized using 1000 steps of steepest descent and 1500 steps of conjugate gradient minimization. Subsequently, the systems were heated to 360 K for further relaxation of the system and equilibrated at the temperature for 200 ps before they were cooled down to 300 K.

Finally, 50 ns production runs were performed at 300 K using Langevin dynamics with a friction coefficient of 5 $ps^{-1}$. The pressure was adjusted to 1 atm by the Berendsen weak-coupling algorithm[15] with a relaxation time constant of 2.0 ps. All dynamic runs used an integral time step of 2 fs. Periodic boundary conditions were used and long-range electrostatic interactions were treated by the particle-mesh Ewald (PME)[16] with a direct space cutoff of 10.0 Å. The SHAKE algorithm[17] was employed to restrain all covalent bonds involving hydrogen atoms.

## 8. Overlapping area calculation

The overlapping area ($S_{OA}$) of each dinucleotide base step of all the structures from crystallization were calculated using *3DNA*[18] directly. These contain the crystal structures GRE-GREDBD, smGRE-GRDBD, mmGRE-GRDBD, and 30 free B-form DNA structures from the PDB database (Supplementary Table S2). The average $S_{OA}$ of each dinucleotide base step of all the complexes from MD simulations were calculated using *3DNA*. These complexes contain GRE-GRDBD, smGRE-GRDBD, mmGRE-

GRDBD, E-box-BCDBD, emE-box-BCDBD, mmE-box-BCDBD, ERE-ERDBD, smERE-ERDBD and mmERE-ERDBD. The $S_{OA}$ distribution of all the ten base pair steps were analyzed statistically from MD simulations of all naked DNAs, all of which were calculated by *3DNA*. These naked DNAs contain GRE, smGRE, mmGRE-GRDBD, E-box, emE-box, mmE-box, ERE, smERE, mmERE and 5′-CG(CG)$_{13}$CG-3′.

## 9. BisChIP-seq and data analysis

*Bmal1*, *clock* and *nr3c* (GR) genes, which were kindly provided by Prof. Jiahuai Han Lab at the Xiamen University, were subcloned into the pCMV14 vector with the 3X Flag-tag at the C-terminus. GR-pCMV14 and DNMT3A-pLX304 (kindly provided by Prof. Zhengfan Jiang lab at Peking University. Because the non-CpG methylation in somatic cells are always low, and the expression of DNMT3A in HEK293T cell is also very low, so we added this vector to increase the non-CpG methylation of HEK293T cell's chromatin.) vectors were then co-transfected into HEK-293T cells using the Genscort II transfection reagent (Wisegen, Nanjing, China), and Dexamethasone (Sigma-Aldrich) was added to a final concentration of 4 nM after 24 h to facilitate the transfer of GR into nucleus. BMAL1-pCMV14, CLOCK-pCMV14 and DNMT3A-pLX304 vectors were also co-transfected into HEK-293T cells, but without the addition of dexamethasone. Cells were collected after 24 h of incubation, followed by the BisChIP-seq experiment that was performed as described previously[19,20].

Cells were cross-linked with 1% formaldehyde for 8 min at room temperature and glycine was added to stop the reaction at a final concentration of 0.125 M. Crosslinked

cells were then washed with phosphate buffered saline several times before resuspension in lysis buffer (1% SDS, 50 mM Tris-HCl, pH8.0, 10 mM EDTA) on ice for 5 min. The lysate was aspirated into a Covaris tube and sonicated by a Covaris S2 (Covaris) with a scheduled program (Duty Cycle: 5%, Intensity: 4, Cycles: 200, Time: 60 s, Total time: 6 min). Subsequently, the lysate was diluted in RIPA buffer (0.5 mM EGTA, 140 mM NaCl, 10 mM Tris-HCl, pH 7.5, 1% Triton-X100, 0.1% SDS, 1% EDTA) and then mixed with Dynabeads Protein G (Life Technologies, NY, USA) for immunoprecipitation, which were sealed with the Anti-Flag antibody (Sigma-Aldrich) at 4 ℃ overnight. The beads were then ligated with target proteins and DNA were washed with the RIPA buffer several times and resuspended in Complete Elution buffer (1% SDS, 20 mM Tris-HCl, pH 7.5, 200 ug/ml proteinase K, 5 mM EDTA, 50 mM NaCl) at 68 ℃ for 2 h. After the digestion by proteinase K, beads were removed by a magnetic shelf and samples were mixed with the same volume of phenol-chloroform-isoamyl alcohol (25:24:1) (Sigma-Aldrich) to extract DNA. After centrifugation, the supernatant was aspirated and mixed with the same volume of chloroform-isoamyl alcohol (24:1) (Amresco) to remove phenol and remained proteins following centrifugation. Next, 100% cold ethanol and linear polyacrylamide(LPA) were mixed with the supernatant to facilitate DNA precipitation with samples left at –80 ℃ for > 1 h. The supernatant was discarded after centrifugation and the DNA pellet collected. The DNA pellet was washed with 70% cold ethanol and the pellet dried.

The DNA library was carried out using a NEBNext DNA Library Prep Master Mix Set for Illumina kit (NEB). After end repair and dA-tailing, methylated adaptors

(NEBNext Multiplex Oligos for Illumina) were applied to ligate with DNA followed by bisulfite conversion using the EZ DNA Methylation kit (ZYMO). DNA were then recovered using AMPure XP beads (Beckman Coulter) after PCR amplification and sequenced by Hiseq2000 (Illumina). Generally, we can acquire 50 ng of DNA from about $10^6$ cells with GR or BMAL1-CLOCK. We also carried out two control experiments, one is the negative control experiments for each sample using a non-specific antibody IgG to confirm that the DNA for sequencing were ChIPed by our target protein; the other is the normal ChIP-seq control experiments without bisulfite conversion step to confirm that the ChIPed sequences in BisChIP-seq experiments is significant and specific. A typical result of the BisChIP-seq, ChIP-seq and non-specific antibody ChIPed signal within a same region were presented in Supplementary Fig. S14 for example.

We sequenced every sample twice and merged the sequences into one dataset before data processing. Bismark[21] was used to align the sequencing data to the human genome hg18 and the methylated cytosine sites were calculated. Since the average insert size of all samples was less than ~202 bp, some of the sequencing from the two ends included overlapped sequencing results and thus a no_overlap process was used to eliminate double reads in the bismark_methylation_extractor step. PCR redundant data were removed by samtools rmdup. The subsequent data screening and statistics were performed by programs written in Perl. We only selected data that were sequenced 4 or more times and calculated the methylation level for each ChIPed cytosine by methylated reads/total reads.

We screened the GRE-half sequence “AGAACA” from the GR ChIPed data and E-box sequence “CACGTG” from the BAML1-CLOCK ChIPed data. We then calculated the distance between these sequences to the nearest transcriptional start site (TSS), which was generated from the gene database based on hg18. Since we only paid attention to the transcriptional regulation of methylation, we then selected the ChIPed sites that were within 100 kb to the nearest TSS.

**10. Methylome data analysis**

Methylation level of “AGAACA”, “CACGTG” and “AGGTCA” sequences in the whole genome bisulfite sequencing data of 35-day old human middle frontal gyrus (tissue), 53-year old female and 55-year old male human dorsal prefrontal cortices (neuron)[22] were analyzed. “AGAACA” is a typical hexametric half site of GRE, which is very conserved for GR binding[4,23]. “CACGTG” is a typical E-box sequence, which can be bound with BMAL1-CLOCK complex[2]. “AGGTCA” sequence “AGGTCA” is a typical hexametric half site of ERE, which is very conserved for ER binding[24]. All the results were obtained by analyzing the Ryan Lister et. al.’s whole-genome 5mC sequencing data [22] using Perl programs written ourselves.

## Supplementary References


1. Jin, J., Lian, T., Xie, X. S. & Su, X.-D. High-accuracy mapping of protein binding stability on nucleosomal DNA using a single-molecule method. *J. Mol. Cell Biol.* **6,** 438–40 (2014).

2. Wang, Z., Wu, Y., Li, L. & Su, X.-D. Intermolecular recognition revealed by the complex structure of human CLOCK-BMAL1 basic helix-loop-helix domains with E-box DNA. *Cell Res.* **23,** 213–24 (2012).

3. Kim, S. *et al.* Probing Allostery Through DNA. *Science (80-. ).* **339,** 816–819 (2013).

4. Meijsing, S. H. *et al.* DNA binding site sequence directs glucocorticoid receptor structure and activity. *Science (80-. ).* **324,** 407–10 (2009).

5. Kabsch, W. XDS. *Acta Crystallogr. D. Biol. Crystallogr.* **66,** 125–32 (2010).

6. Vagin, A. & Teplyakov, A. Molecular replacement with MOLREP. *Acta Crystallogr. D. Biol. Crystallogr.* **66,** 22–5 (2010).

7. Collaborative Computing Project. The CCP4 suite: programs for protein crystallography. *Acta Crystallogr. D. Biol. Crystallogr.* **50,** 760–3 (1994).

8. Emsley, P. & Cowtan, K. Coot: model-building tools for molecular graphics. *Acta Crystallogr. Sect. D Biol. Crystallogr.* **60,** 2126–2132 (2004).

9. Adams, P. D. *et al.* PHENIX: a comprehensive Python-based system for macromolecular structure solution. *Acta Crystallogr. D. Biol. Crystallogr.* **66,** 213–21 (2010).

10. Chen, V. B. *et al.* MolProbity: all-atom structure validation for macromolecular crystallography. *Acta Crystallogr. D. Biol. Crystallogr.* **66,** 12–21 (2010).

11. Case, D. A. *et al.* AMBER 11. *Univ. California, San Fr.* (2010).

12. Berendsen, H. J. C., Grigera, J. R. & Straatsma, T. P. The missing term in effective pair potentials. *J. Phys. Chem.* **91,** 6269–6271 (1987).

13. Pérez, A. *et al.* Refinement of the AMBER force field for nucleic acids: improving the description of alpha/gamma conformers. *Biophys. J.* **92,** 3817–29 (2007).

14. Aduri, R. *et al.* AMBER Force Field Parameters for the Naturally Occurring Modified Nucleosides in RNA. *J. Chem. Theory Comput.* **3,** 1464–1475 (2007).

15. Berendsen, H. J. C., Postma, J. P. M., van Gunsteren, W. F., DiNola, A. & Haak, J. R. Molecular dynamics with coupling to an external bath. *J. Chem. Phys.* **81,** 3684 (1984).

16. Darden, T., York, D. & Pedersen, L. Particle mesh Ewald: An N· log(N) method for Ewald sums in large systems. *J. Chem. Phys.* **98,** 10089 (1993).

17. Ryckaert, J.-P., Ciccotti, G. & Berendsen, H. J. . Numerical integration of the cartesian equations of motion of a system with constraints: molecular dynamics of n-alkanes. *J. Comput. Phys.* **23,** 327–341 (1977).

18. Lu, X.-J. & Olson, W. K. 3DNA: a software package for the analysis, rebuilding and visualization of three-dimensional nucleic acid structures. *Nucleic Acids Res.* **31,** 5108–21 (2003).

19. Statham, A. L. *et al.* Bisulfite sequencing of chromatin immunoprecipitated DNA (BisChIP-seq) directly informs methylation status of histone-modified DNA. *Genome Res.* **22,** 1120–7 (2012).

20. Brinkman, A. B. *et al.* Sequential ChIP-bisulfite sequencing enables direct genome-scale investigation of chromatin and DNA methylation cross-talk. *Genome Res.* **22,** 1128–38 (2012).

21. Krueger, F. & Andrews, S. R. Bismark: a flexible aligner and methylation caller for Bisulfite-Seq applications. *Bioinformatics* **27,** 1571–2 (2011).

22. Lister, R. *et al.* Global epigenomic reconfiguration during mammalian brain development. *Science* **341,** 1237905 (2013).

23. Luisi, B. F. *et al.* Crystallographic analysis of the interaction of the glucocorticoid receptor with DNA. *Nature* **352,** 497–505 (1991).

24. Schwabe, J. W., Chapman, L., Finch, J. T. & Rhodes, D. The crystal structure of the estrogen receptor DNA-binding domain bound to DNA: how receptors discriminate between their response elements. *Cell* **75,** 567–78 (1993).

## Supplementary Figures

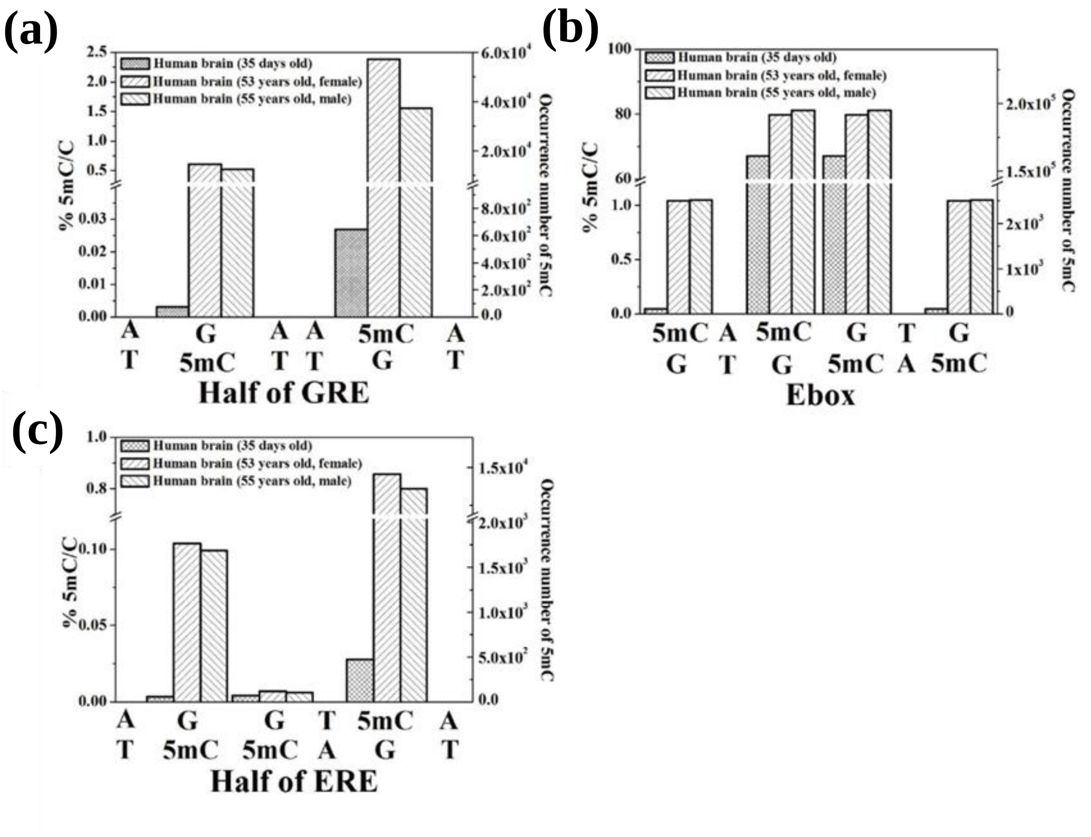


**Supplementary Figure S1:**

**Methylation level analysis of candidates for TFs' binding in human brain. (a).** Methylation level analysis of "AGAACA" sequence in the whole genome of one fetal brain neuron and two old adults' neurons, "AGAACA" is a hexametric half site of GRE, which is a strong candidate for GR binding[4,23]. **(b).** Methylation level analysis of E-box in the whole genome of one fetal brain neuron and two old adults' neurons, E-box is a protein-binding site, which can be bound with BMAL1-CLOCK complex to regulate gene expression in neurons[2]. **(c).** Methylation level analysis of "AGGTCA" sequence in the whole genome of one fetal brain neuron and two old adults' neurons, "AGGTCA" is a hexametric half site of ERE, which is a strong candidate for ER binding[24]. And the occurrence No. of methylated sites are also demonstrated. There are tens to thousands binding sites with mCH in the brain neurons all in these three TFs' binding candidates, and the proportions of mCH were increased when the brain was developed from the fetal to adult, except the latter cytosine of CC context in ERE. The methylation level of mCG in E-box is pretty high and increased from the fetal to adult too. All the results were obtained by analyzing the Ryan Lister et. al.'s whole-genome 5mC sequencing data[22].

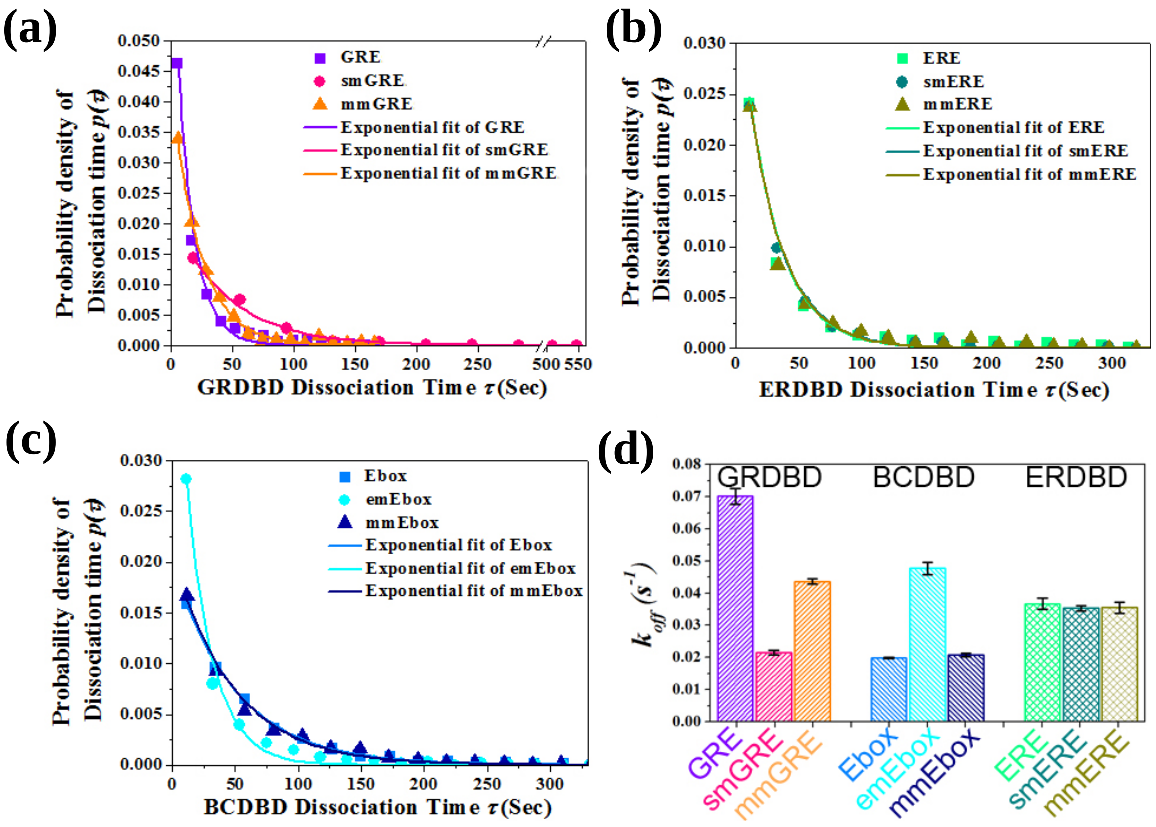


**Supplementary Figure S2:**

**$k_{off}$ result of transcription factors GRDBD/ERDBD/BCDBD**

**(a)** The single molecule dissociation curve of GRDBD and its target site GREs with different cytosine methylated. **(b)** The single molecule dissociation curve of ERDBD and its target site EREs with different cytosine methylated. **(c)** The single molecule dissociation curve of BCDBD and its target site E-boxes with different cytosine methylated. **(d)** Single molecule dissociation curve fitted $k_{off}$ of three transcription factors and its target sites with different methylated states.

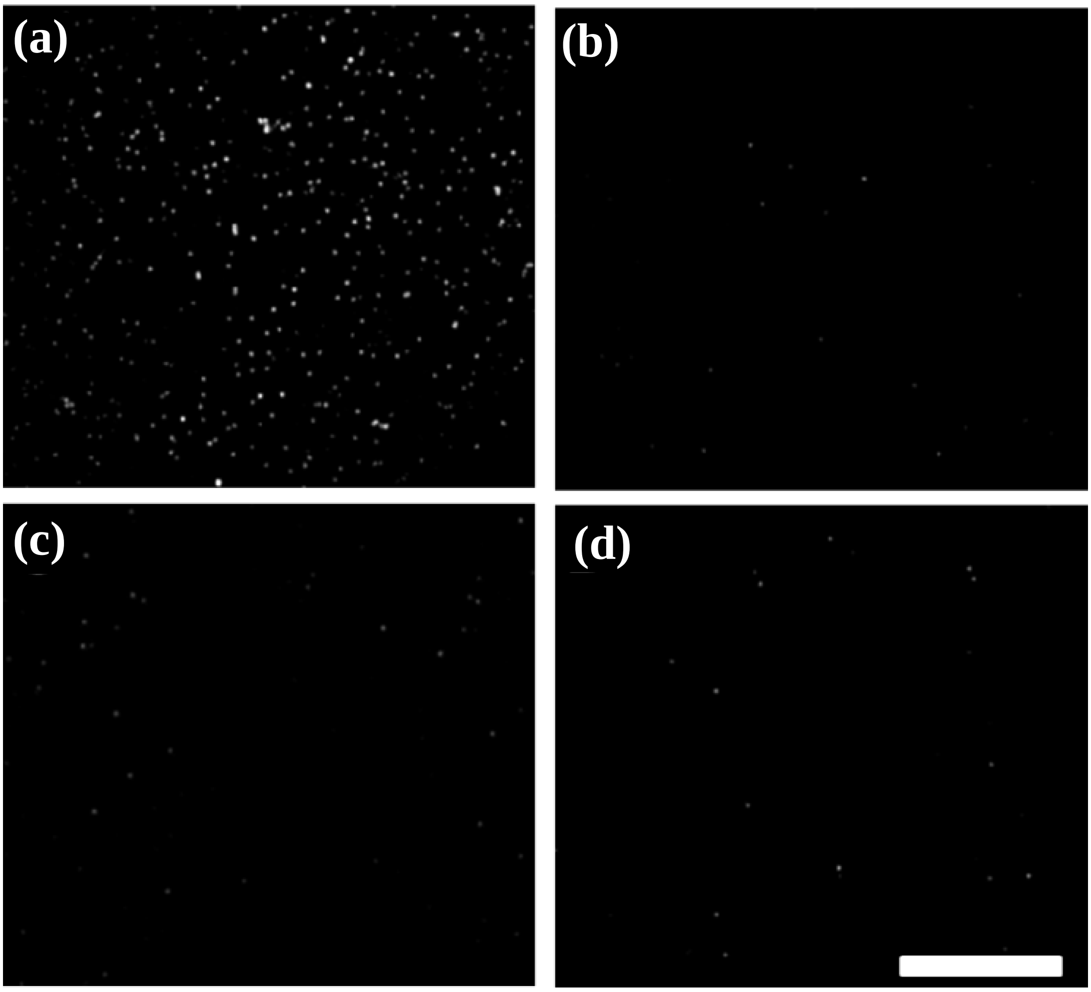


**Supplementary Figure S3:**

(a) TIRF images for GRDBD binding to GRE within the immobilized DNA before GRDBD dissociating. (b). TIRF images for GRDBD binding to GRE within the immobilized DNA after GRDBD dissociating. (c) TIRF images for GRDBD non-specific binding to surface without immobilized DNA. (d) TIRF images for GRDBD non-specific binding to the immobilized DNA without GRE. Scale bar, 30μm.

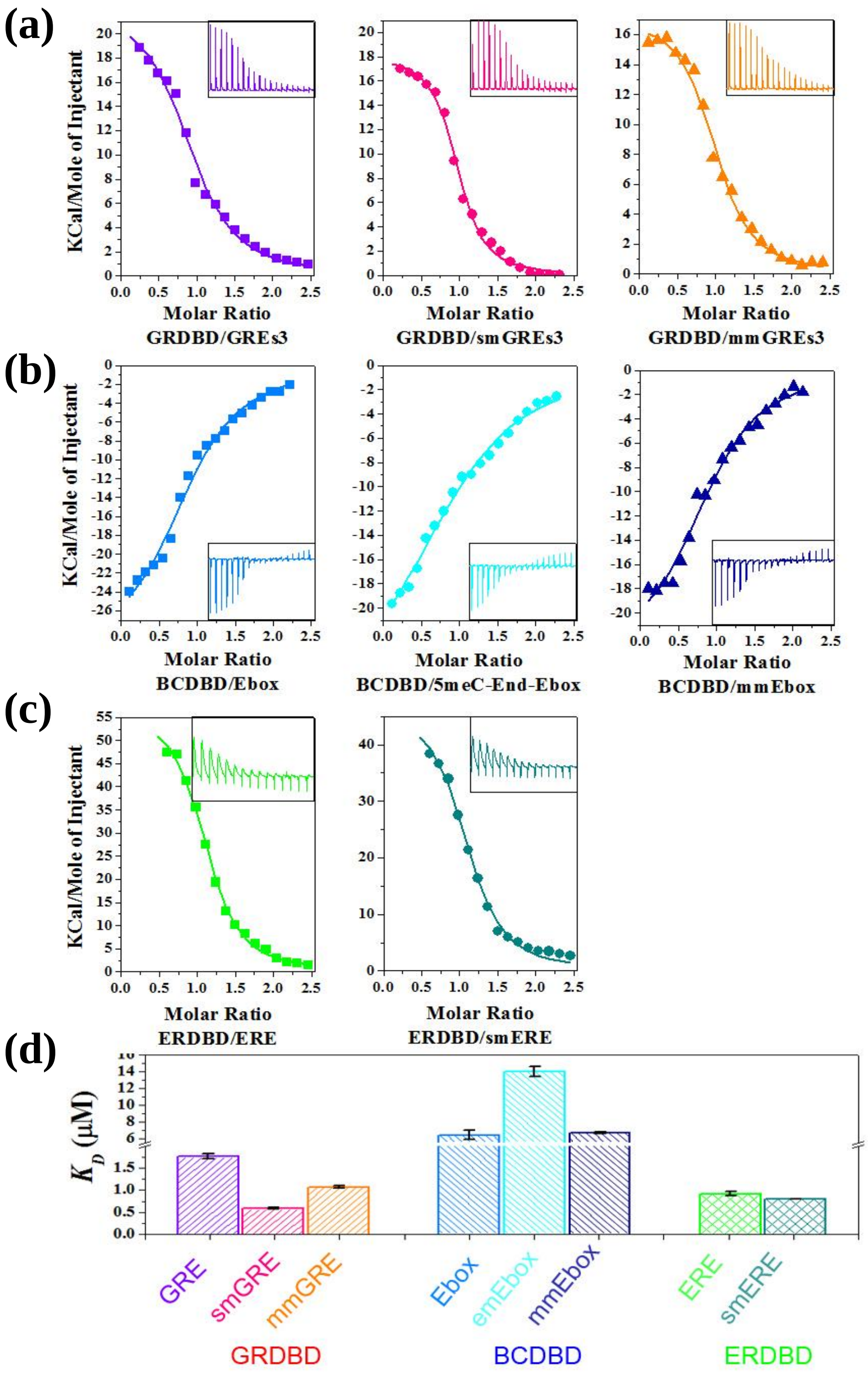


**Supplementary Figure S4:**

***K<sub></sub>***

**$K_D$ result of transcription factors GRDBD/ERDBD/BCDBD**

**(a)(b)(c)** ITC titration result and fitted curves of the three transcription factors GRDBD, ERDBD and BCDBD **(d)** $K_D$ calculated from the ITC fitted curves of the three transcription factors and targets sites with different cytosine methylated.

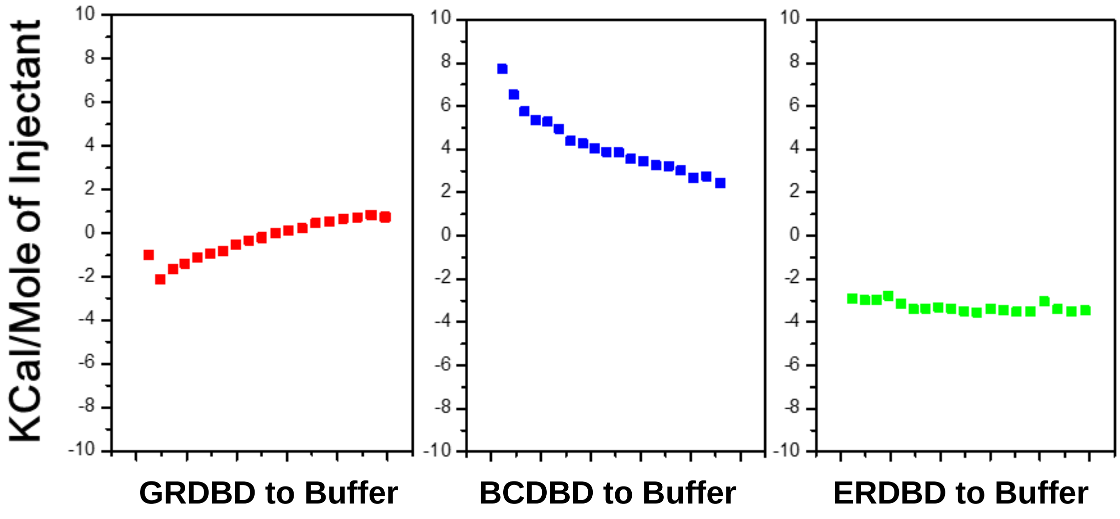


**Supplementary Figure S5:**

Control experiments for ITC, which the proteins (0.2 mM GRDBD, 0.2 mM ERDBD, and 0.4 mM BCDBD) were titrated into the buffers only (buffer 2 for GRDBD and BCDBD, and buffer 3 for ERDBD).

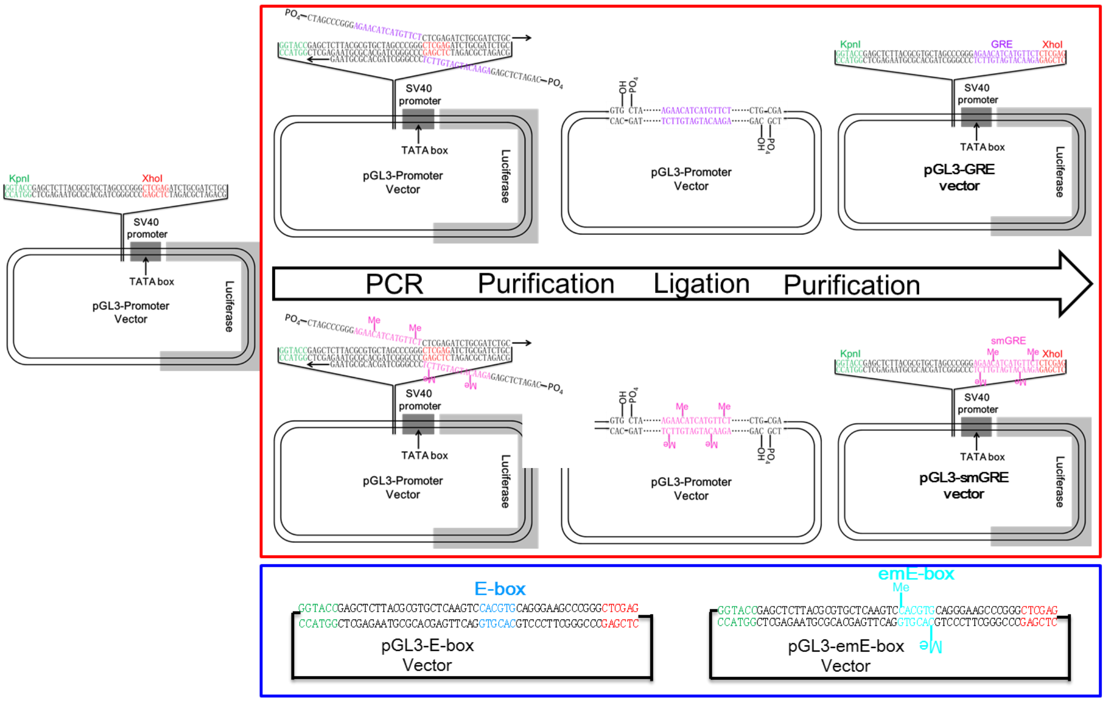


**Supplementary Figure S6:**

Illustration of preparation of unmethylated and methylated plasmids, which were used in the cell-based luciferase assay.

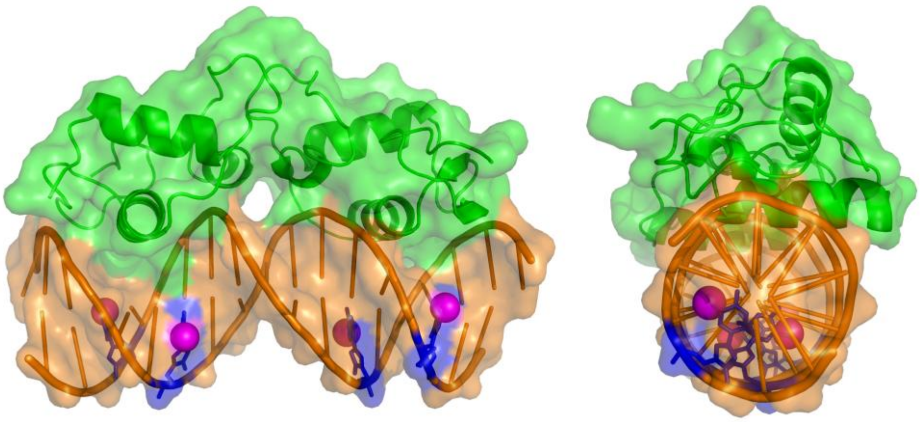

**Supplementary Figure S7:**

Overall structure of smGRE-GRDBD, shown as cartoon and surface. The green part represents GRDBD, and the orange part is smGRE. The blue stick represents methylated cytosine, and the methyl group is shown as magenta sphere.

(a)

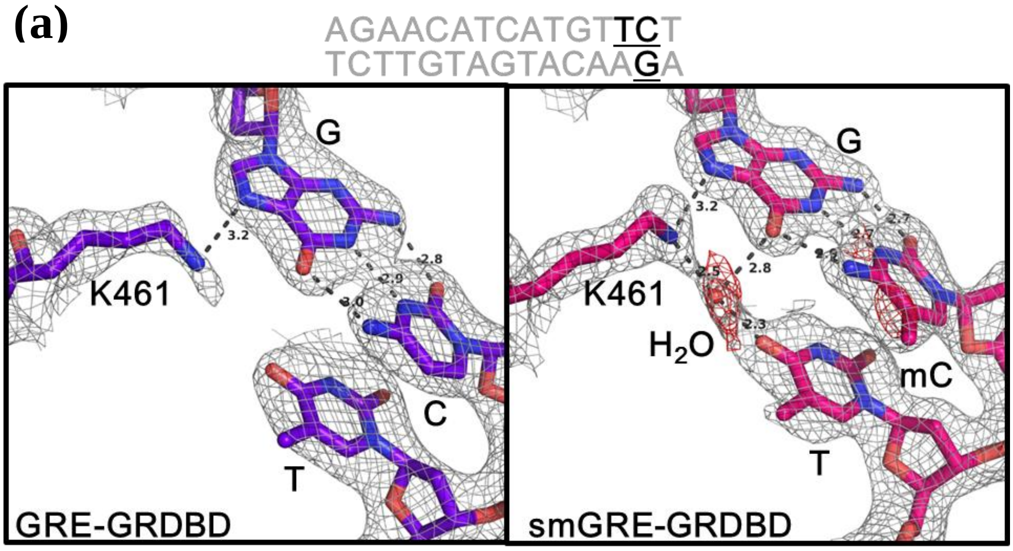


(b)

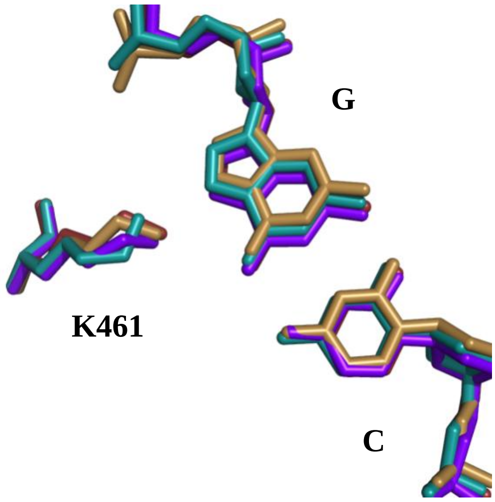


**Supplementary Figure S8:**

(a) The electron density map (1.0 σ) is shown in gray and the omit map (indicating the water molecule and methyl of mC, 3.0 σ) is shown in red. New hydrogen bonds between K461 of GRDBD and both mC-paired guanine and thymine (AGAACATCATGTTCT) were produced through a well-ordered water molecule when the cytosine was methylated in the smGRE-GRDBD complex. (b) K461 of GRDBD interacting with guanine within sequence "TGTTCT" in crystal structures 4RCQ (our unmethylated GRE-GRDBD complex, purple), 3G6P (deep teal), 3G6R (Blue) and 3G9J (Sand). K461 only forms one hydrogen bond with the guanine in all unmethylated GRE-GRDBD complexes crystal structures whose GRE has AGAACA(TGTTCT) sequences, even if the other half sequence of GRE has been changed.

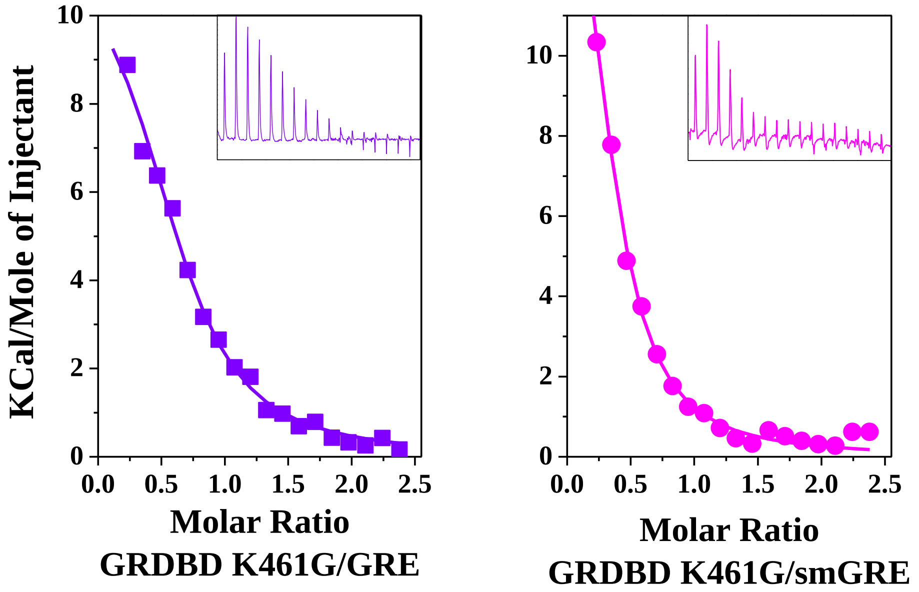


**Supplementary Figure S9:**

ITC titration result and fitted curves of mutant K461G of GRDBD binding to GRE(left) and smGRE (right).

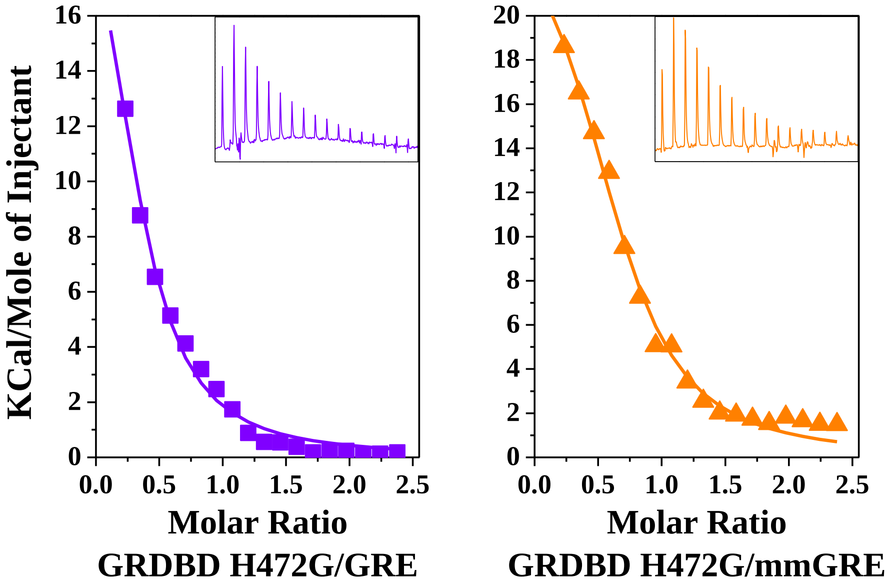


**Supplementary Figure S10:**

ITC titration result and fitted curves of mutant H472G of GRDBD binding to GRE (left) and mmGRE (right).

(a)

(b)

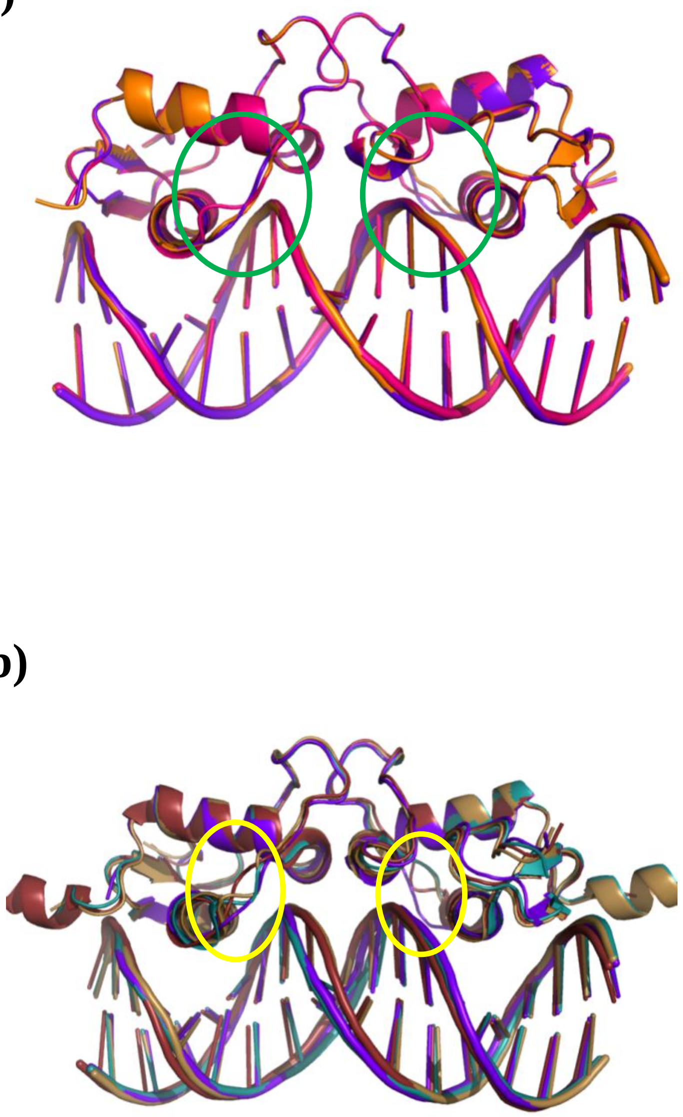

**Supplementary Figure S11:**
(a) Alignment of complexes GRE-GRDBD (5EMQ, purple), smGRE-GRDBD (5EMC, magenta) and mmGRE-GRDBD (5EMP, orange). (b) Alignment of complexes GRE-GRDBD (5EMQ, purple), 3G6P (deep teal), 3G6R (blue) and 3G9J (sand). The lever arm loops are circled.

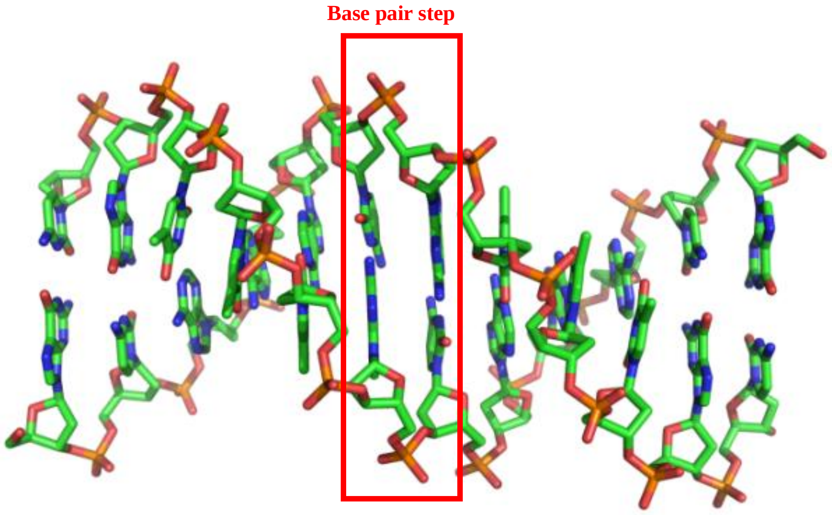


**Supplementary Figure S12:**
The illumination of base pair step.

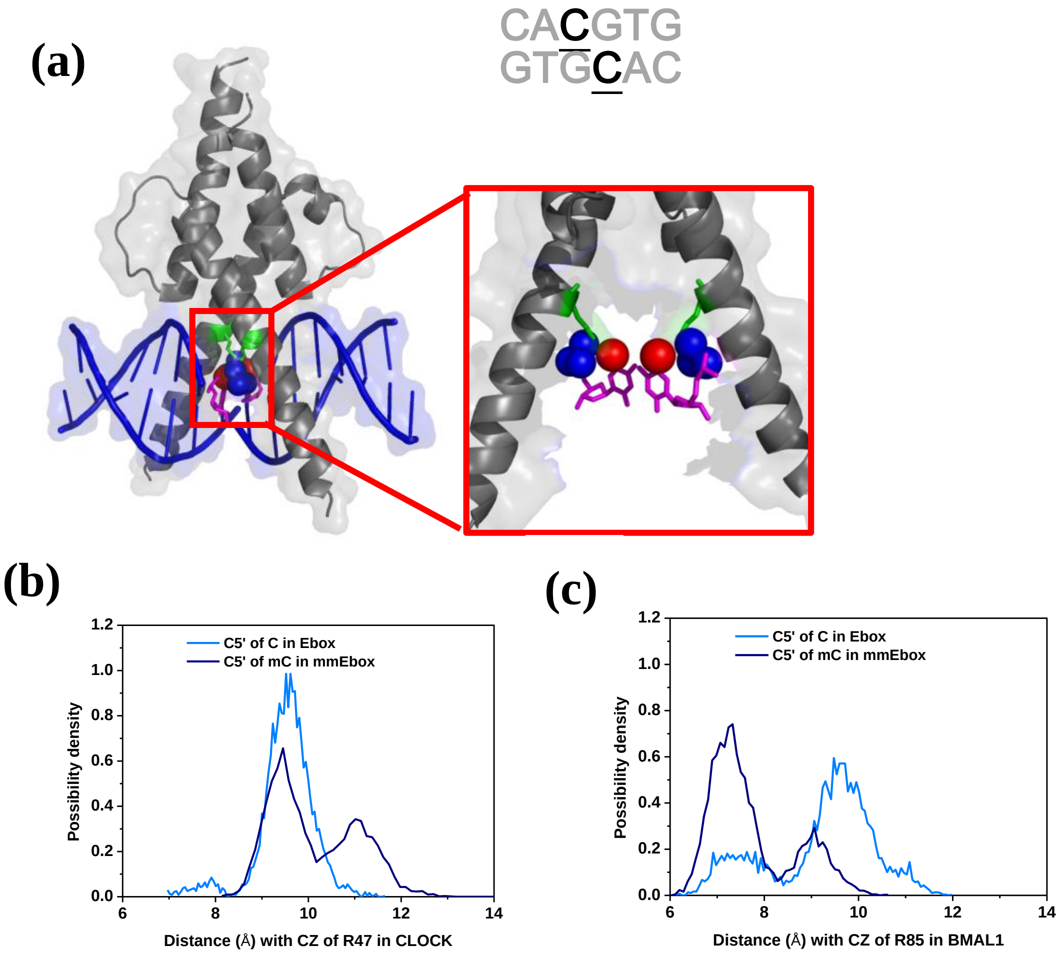


**Supplementary Figure S13:**

(a) The structure of mmE-box-BCDBD complex mutated from 4H10. The corresponding cytosines were directly mutated into methylated cytosines. Green residues represent R85 of BMAL1 and R47 of CLOCK. Blue spheres represent their terminal amino groups. Mutated cytosines were shown as magenta cartoon and the red sphere represents its methyl group. The methyl group is far away from the amino groups, so there is no resistance in this structure. (**b**) The distribution of the distance between the carbon atom $C_Z$ of CLOCK R47 and the carbon atom C5 of the cytosine (CACGTG) in Ebox and mmEbox. (**c**) The distribution of the distance between the carbon atom $C_Z$ of BMAL1 R85 and the carbon atom C5 of the cytosine (CACGTG) in Ebox and mmEbox. Data in (**b)** and (**c)** come from MD simulations.

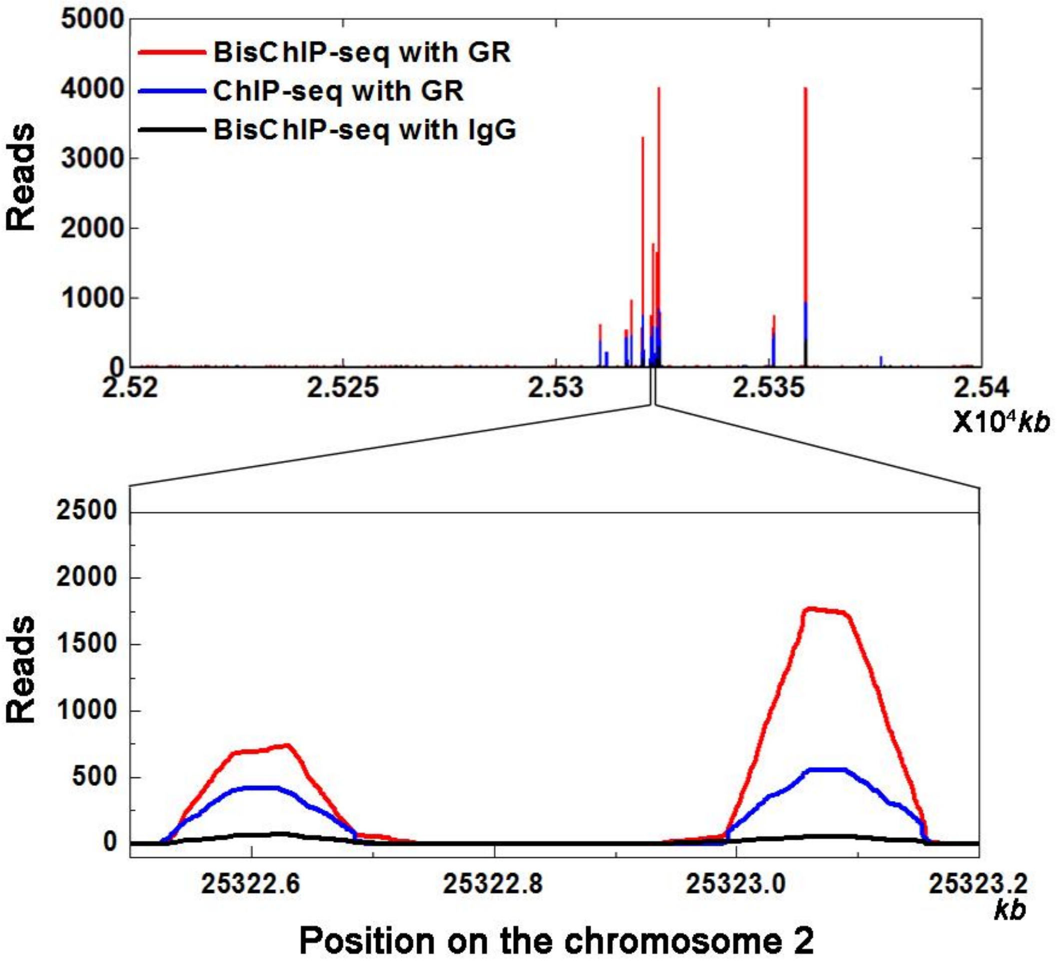


**Supplementary Figure S14:**

A typical result of the BisChIP-seq (with GR), normal ChIP-seq (with GR) and non-specific antibody (IgG) ChIPed signal within a same region, there is an "AGAACA" sequence at position 25323059-25323064. Reads is the sequencing depth of each position. Because the total amount of sequencing of normal ChIP-seq is half of that of BisChIP-seq or IgG ChIP-seq, the average sequencing depth of normal ChIP-seq is less than that of BisChIP-seq. Both the peak range and width indicated that the BisChIP-seq assay has worked well.

## Supplementary Tables

## Supplementary Table S1:

**Data collection and refinement statistics.**

| | smGRE-GRDBD | GRE-GRDBD | mmGRE-GRDBD |
|---|---|---|---|
| **Wavelength (Å)** | 0.98 | 1.10 | 1.10 |
| **Resolution range (Å)** | 37.26 - 2.3 | 37.36 - 2.3 | 35.01 - 2.3 |
| **Space group** | P $2_1$ $2_1$ $2_1$ | P $2_1$ $2_1$ $2_1$ | P $2_1$ $2_1$ $2_1$ |
| **Unit cell** | 38.94 99.64 112.23<br>90 90 90 | 39.03 100.38 111.90<br>90 90 90 | 39.28 100.49 112.04<br>90 90 90 |
| **Total reflections** | 76953 | 56936 | 104716 |
| **Unique reflections** | 19812 (1978) | 19855 (1971) | 20343 (1995) |
| **Redundancy** | 3.88 | 2.87 | 5.15 |
| **Completeness (%)** | 98.37 (100.00) | 97.92 (99.50) | 99.46 (99.95) |
| **Mean I/sigma(I)** | 6.37 (2.32) | 10.99 (2.56) | 11.73 (2.90) |
| **R-factor** | 0.2248 (0.2844) | 0.2000 (0.2638) | 0.1927 (0.2444) |
| **R-free** | 0.2609 (0.3371) | 0.2251 (0.3025) | 0.2380 (0.2810) |
| **Number of atoms** | 1986 | 1839 | 1865 |
| **macromolecules** | 1792 | 1800 | 1814 |
| **$R_{mer}$ （%）** | 15.1/62.2 | 7.9/55.0 | 9.3/55.2 |
| **RMS(bonds)** | 0.006 | 0.009 | 0.038 |
| **RMS(angles)** | 0.92 | 1.32 | 1.52 |
| **Ramachandran favored (%)/outliers(%)** | 94.2/0.7 | 94.9%/0 | 96.4/0.7 |
| **Average B-factor** | 41.60 | 47.90 | 49.70 |

**Supplementary Table S2:**

**Crystal structures of free B-form DNA in PDB data base, which we used to calculate the overlapping area ($S_{OA}$) of ten nucleotide base pair steps.（NDB: Nucleic Acid Database/ http://ndbserver.rutgers.edu/）**

| PDB ID | DNA sequence |
|---|---|
| 1DCV | 5’-CCGCTAGCGG-3’/ 5’-CCGCTAGCGG-3’ |
| 1EHV | 5’-CGCGAATTCGCG-3’/ 5’-CGCGAATTCGCG-3’ |
| 1HQ7 | 5’-GCAAACGTTTGC-3’/ 5’-GCAAACGTTTGC-3’ |
| 1ILC | 5’-ACCGAATTCGGT-3’/ 5’-ACCGAATTCGGT-3’ |
| 1N4E | 5’-GCTTAATTCG-3’/ 5’-CGAATTAAGC-3’ |
| 1VTD | 5’-ACCGGCGCCACA-3’/ 5’-TGTGGCGCCGGT-3’ |
| 1WQY | 5’-CCATTAATGG-3’/ 5’-CCATTAATGG-3’ |
| 1WQZ | 5’-CCATTAATGG-3’/ 5’-CCATTAATGG-3’ |
| 1ZF5 | 5’-CCAGCGCTGG-3’/ 5’-CCAGCGCTGG-3’ |
| 1ZFC | 5’-CCGATATCGG-3’/ 5’-CCGATATCGG-3’ |
| 1ZFE | 5’-CCTGCGCAGG-3’/ 5’-CCTGCGCAGG-3’ |
| 1ZFF | 5’-CCGAATTCGG-3’/ 5’-CCGAATTCGG-3’ |
| 1ZFG | 5’-CCGAGCTCGG-3’/ 5’-CCGAGCTCGG-3’ |
| 1ZFH | 5’-CCTAATTAGG -3’/ 5’-CCTAATTAGG -3’ |
| 1ZFM | 5’-CCGCTAGCGG-3’/ 5’-CCGCTAGCGG-3’ |
| 2AF1 | 5’-CGATATATATAT-3’/ 5’-CGATATATATAT-3’ |
| 2G1Z | 5’-AAATTT-3’/ 5’-AAATTT-3’ |
| 3L1Q | 5’-TGGCCTTAAGG-3’/ 5’-TGGCCTTAAGG-3’ |
| 4J2I | 5’-AATAAATTTATT-3’/ 5’-AATAAATTTATT-3’ |
| 194D | 5’-CGCGTTAACGCG-3’/ 5’-CGCGTTAACGCG-3’ |
| 237D | 5’-CGCAATTGCG-3’/ 5’-CGCAATTGCG-3’ |
| 250D | 5’-CGCTAGCG-3’/ 5’-CGCTAGCG-3’ |
| 251D | 5’-CTCGAG-3’/ 5’-CTCGAG-3’ |
| 252D | 5’-CGCAATTGCG -3’/ 5’-CGCAATTGCG -3’ |
| 287D | 5’-CGCGATATCGCG-3’/ 5’-CGCGATATCGCG-3’ |
| 307D | 5’-CAAAGAAAAG-3’/ 5’-CTTTTCTTTG-3’ |
| 309D | 5’-CGACGATCGT-3’/ 5’-CGACGATCGT-3’ |
| 330D | 5’-ACCGCCGGCGCC-3’/ 5’-GGCGCCGGCGGT-3’ |
| 424D | 5’-ACCGACGTCGGT-3’/ 5’-ACCGACGTCGGT-3’ |
| 425D | 5’-ACCGGTACCGGT-3’/ 5’-ACCGGTACCGGT-3’ |

**Supplementary Table S3 :**

**DNA for single molecule assay**

| Binding site | Sequence* |
|---|---|
| GRE_SM | 5’-AGAACATCATGTTCT-3’ |
| | 3’-TCTTGTAGTACAAGA-5’ |
| smGRE_SM | 5’-AGAAmCATCATGTTmCT-3’ |
| | 3’-TmCTTGTAGTAmCAAGA-5’ |
| mmGRE_SM | 5’-AGAACATmCATGTTCT-3’ |
| | 3’-TCTTGTAGTACAAGA-5’ |
| E-box_SM | 5’-CACGTG3’ |
| | 3’-GTGCAC-5’ |
| emE-box_SM | 5’-mCACGTG-3’ |
| | 3’-GTGCAmC-5’ |
| mmE-box_SM | 5’-CAmCGTG-3’ |
| | 3’-GTGmCAC-5’ |
| ERE_SM | 5’-AGGTCACAGTGACCT-3’ |
| | 3’-TCCAGTGTCACTGGA-5’ |
| smERE_SM | 5’-AGGTmCACAGTGACmCT-3’ |
| | 3’-TmCCAGTGTCAmCTGGA-5’ |
| mmERE_SM | 5’-AGGTCAmCAGTGACCT-3’ |
| | 3’-TCCAGTGTmCACTGGA-5’ |

* Full length sequence (here only present the forward strand, the reverse strand hasn’t biotin):

GRE&ERE：

Biotin-TGTGGAAACCGCATTCTGTACTTTCGTGCTGTCCC+ Binding site + GAGGCGGAT

E-box：

Biotin-TGTGGAAACCGCATTCTGTACTTTCGTGCTGTCGGAA+ Binding site + ACCCGCGGAT

**Supplementary Table S4:**
**DNA for ITC assay**

| DNA | Sequence |
|---|---|
| GRE_ITC | 5’-CCAGAACATCATGTTCTGAG-3’ |
| | 3’-GGTCTTGTAGTACAAGACTC-5’ |
| smGRE_ITC | 5’-CCAGAAmCATCATGTTmCTGAG-3’ |
| | 3’-GGTmCTTGTAGTAmCAAGACTC-5’ |
| mmGRE_ITC | 5’-CCAGAACATmCATGTTCTGAG-3’ |
| | 3’-GGTCTTGTAGTACAAGACTC-5’ |
| E-box_ITC | 5’-GGAACACGTGACCC-3’ |
| | 3’-CCTTGTGCACTGGG-5’ |
| emE-box_ITC | 5’-GGAAmCACGTGACCC-3’ |
| | 3’-CCTTGTGCAmCTGGG-5’ |
| mmE-box_ITC | 5’-GGAACAmCGTGACCC-3’ |
| | 3’-CCTTGTGmCACTGGG-5’ |
| ERE_ITC | 5’-CCAGGTCACAGTGACCTGAG-3’ |
| | 3’-GGTCCAGTGTCACTGGACTC-5’ |
| smERE_ITC | 5’-CCAGGTmCACAGTGACmCTGAG-3’ |
| | 3’-GGTmCCAGTGTCAmCTGGACTC-5’ |
| mmERE_ITC | 5’-CCAGGTCAmCAGTGACCTGAG-3’ |
| | 3’-GGTCCAGTGTmCACTGGACTC-5’ |

**Supplementary Table S5:**

**DNA for cell-based luciferase assay**

| DNA | Sequence* |
|---|---|
| GRE-F-primer | $PO_4$-CTAGCCCGGGAGAACATCATGTTCTCTCGAGATCTGCGATCTGC |
| GRE-R-primer | $PO_4$-CAGATCTCGAGAGAACATGATGTTCTCCCGGGCTAGCACGCGTAAG |
| smGRE-F-primer | $PO_4$-CTAGCCCGGGAGAAmCATCATGTTmCTCTCGAGATCTGCGATCTGC |
| smGRE-R-primer | $PO_4$-CAGATCTCGAGAGAAmCATGATGTTmCTCCCGGGCTAGCACGCGTAAG |
| E-box-F- primer | PO4-CTTACGCGTGCTCAAGTCCACGTGCAGGGAAGCCCGGGCTCGAGATC TGCGATCTGC |
| E-box-R- primer | PO4-CGAGCCCGGGCTTCCCTGCACGTGGACTTGAGCACGCGTAAGAGCTCGGTACCTATCG |
| emE-box-F- primer | $PO_4$-CTTACGCGTGCTCAAGTCmCACGTGCAGGGAAGCCCGGGCTCGAGATC TGCGATCTGC |
| emE-box-R- primer | $PO_4$-CGAGCCCGGGCTTCCCTGmCACGTGGACTTGAGCACGCGTAAGAGCTCGGTACCTATCG |

**Supplementary Table S6:**
**DNA for crystallization**

| DNA | Sequence |
|---|---|
| GRE_Crystal | 5’-CCAGAACATCATGTTCTG -3’ |
| | 3’- GTCTTGTAGTACAAGACC-5’ |
| smGRE_Crystal | 5’-CCAGAAmCATCATGTTmCTG-3’ |
| | 3’- GTmCTTGTAGTAmCAAGACC-5’ |
| mmGRE_Crystal | 5’-CCAGAACATmCATGTTCTG-3’ |
| | 3’- GTCTTGTAGTACAAGACC-5’ |
